# Divergent Regulation of Actin Dynamics and Megakaryoblastic Leukemia-1 and -2 (Mkl1/2) by cAMP in Endothelial and Smooth Muscle Cells

**DOI:** 10.1038/s41598-017-03337-0

**Published:** 2017-06-16

**Authors:** Madeleine C. Smith, Claire A. Hudson, Tomomi E. Kimura, Stephen J. White, Graciela B. Sala-Newby, Andrew C. Newby, Mark Bond

**Affiliations:** 1 School of Clinical Sciences, University of Bristol, Research Floor Level 7, Bristol Royal Infirmary, Bristol, BS2 8HW UK; 2 Manchester Metropolitan University, John Dalton Building, Chester Street, Manchester, M1 5GD UK

## Abstract

Proliferation and migration of vascular smooth muscle cells (VSMCs) or endothelial cell (ECs) promote or inhibit, respectively, restenosis after angioplasty, vein graft intimal thickening and atherogenesis. Here we investigated the effects of cAMP-induced cytoskeletal remodelling on the serum response factor (SRF) co-factors Megakaryoblastic Leukemia-1 and -2 (MKL1 and MKL2) and their role in controlling VSMC and EC proliferation and migration. Elevation of cAMP using forskolin, dibutyryl-cAMP (db-cAMP), BAY60-6583 or Cicaprost induced rapid cytoskeleton remodelling and inhibited proliferation and migration in VSMCs but not EC. Furthermore, elevated cAMP inhibited mitogen-induced nuclear-translocation of MKL1 and MKL2 in VSMCs but not ECs. Forskolin also significantly inhibited serum response factor (SRF)-dependent reporter gene (SRE-LUC) activity and mRNA expression of pro-proliferative and pro-migratory MKL1/2 target genes in VSMCs but not in ECs. In ECs, MKL1 was constitutively nuclear and MKL2 cytoplasmic, irrespective of mitogens or cAMP. Pharmacological or siRNA inhibition of MKL1 significantly inhibited the proliferation and migration of VSMC and EC. Our new data identifies and important contribution of MKL1/2 to explaining the strikingly different response of VSMCs and ECs to cAMP elevation. Elucidation of these pathways promises to identify targets for specific inhibition of VSMC migration and proliferation.

## Introduction

Vascular smooth muscle cell (VSMC) proliferation and migration contribute to restenosis after angioplasty, late vein graft failure and atherosclerosis^1^. Current-generation anti-mitotics, such as rapamycin and paclitaxel, increase thrombotic risk by impairing endothelial cell (EC) regrowth^2^. Clearly, there is a clinical requirement to identify signalling pathways that selectively inhibit VSMC proliferation and migration whilst sparing protective EC functions.

Elevated levels of 3′-5′ cyclic adenosine monophosphate (cAMP), an intracellular second messenger synthesised by receptor-stimulated adenylyl cyclase, potently inhibit VSMC proliferation and migration *in vitro* and *in vivo* ^3–5^ and reduce intimal lesion formation in animal models of vascular injury^6^. Crucially, cAMP also induces vascular protective effects in ECs, where it promotes barrier function^7^, and inhibits inflammation^8^, ROS generation^9^ and monocyte adhesion^10^. It is not surprising, therefore, that altered or aberrant cAMP signalling has been linked to numerous vascular pathologies, including angioplasty restenosis^11^, late vein graft failure^12^ and atherogenesis^13^.

Elevation of cAMP inhibits VSMC proliferation by downregulating multiple key cell-cycle intermediates^4, 14–17^ but the upstream signalling mechanisms are incompletely characterised. The cAMP-activated transcription factor, CREB, has been implicated because forced expression of constitutively-active CREB mutants inhibit VSMC proliferation and migration^18^. However, selective PKA-agonists, which potently activate CREB, are insufficient to inhibit VSMC proliferation, implicating additional cAMP-sensitive pathways. We recently demonstrated that the anti-mitotic activity of cAMP was associated with actin-cytoskeleton remodelling in response to inhibition of members of the Rho GTPases by cAMP^19^. Forced expression of active-mutants of RhoA or Rac1 prevent cAMP-induced growth arrest^19^, whereas RhoA and Rac1 inhibition mimic the effects of cAMP^19, 20^. By contrast, the effects of cAMP on proliferation and migration in ECs are less well defined. Elevated cAMP inhibits RhoA activity also in EC, but the effects on EC proliferation are controversial. Some studies report inhibitory effects^21–23^, whereas others report stimulatory effects^24, 25^.

Moreover, the mechanisms that sense changes in actin polymerisation and ultimately modulate responses in VSMCs and ECs have not been described. Some studies have linked actin dynamics to regulation of the MAPK pathway, proliferation and migration^26–28^ but effects of cAMP on proliferation and migration can be dissociated from cAMP-mediated MAPK inhibition^15^. The present study focusses on the MADS box transcription factor, serum response factor (SRF), which in conjunction with the co-activator, myocardin, modulates numerous smooth muscle specific contractile genes^29^. In NIH-3T3 fibroblasts, increased levels of actin monomer (G-actin) inhibits a specific subset of SRF target genes involved in proliferation and migration^30, 31^. This is mediated not by myocardin but by the related co-factors megakaryoblastic leukemia 1/2 (MKL1 and MKL2)^30, 32^. MKL1 and MKL2 link actin cytoskeleton remodelling to gene transcription via RPEL domains that bind G-actin^33^. In the G-actin bound state, these co-factors are sequestered in the cytoplasm and unable to activate nuclear SRF-dependent transcription^33^. Interestingly, genome wide association studies (GWAS) analysis identified a SNP in the MKL1 promoter that was associated with increased incidence of coronary heart disease^34^ and MKL1 deletion in mice causes a significant reduction in pathological intima formation^35^.

Here, we compare the effects of cAMP on proliferation, migration, actin cytoskeletal remodelling and MKL1/2 activation in VSMCs and ECs. Our data demonstrate opposite effects of cAMP on proliferation and migration of VSMCs compared to ECs, which are related to divergent regulation of actin polymerisation and subsequent MKL-dependent gene transcription. These data show that differential regulation of MKL1/2 contributes to the divergent effects of cAMP in VSMCs and ECs and helps identify targets for selective intervention.

## Results

### Divergent effects of elevated cAMP on VSMC and EC proliferation and migration

We initially compared the effects of elevated cAMP on the proliferation and migration of VSMCs and ECs. The direct adenylate-cyclase activator, forskolin, significantly increased intracellular levels of cAMP in either VSMC or EC, although levels were higher in VSMC (Supplement Fig. 1a and c). We therefore also stimulated cells with a fixed concentration (500 µM) of the cAMP analogue dibutyryl-cAMP. Stimulation of RaVSMCs or HuVSMCs with the adenylate-cyclase activator, forskolin, or the cAMP analogue db-cAMP resulted in a significant inhibition of serum stimulated proliferation, measured by BrdU incorporation (Supplement Fig. 2). In contrast, forskolin or db-cAMP resulted in a significant increase in proliferation of HUVECs and no significant change in HCAEC proliferation (Supplement Fig. 2). Stimulation of RaVSMCs or HuVSMCs with forskolin resulted in a significant inhibition of migration in real-time scratch wound assays (Supplement Fig. 3a,c). Stimulation of RaVMSC with db-cAMP also inhibited migration (Supplement Fig. 3b). However, the effect of db-cAMP on HuVSMC migration was very small and only just significant (Supplement Fig. 3d). In HUVECs and HCAECs, forskolin or db-cAMP did not significantly inhibit migration (Supplement Fig. 4a–d). Forskolin stimulation of HCAECs and db-cAMP stimulation of HUVECs actually resulted in a small delayed increase in wound closure. Activation of the adenosine A2B-receptor or the prostacyclin receptor, which are known to be coupled to adenylate cyclase activation, using BAY60-6583 or Cicaprost respectively, increased intracellular cAMP levels (Supplement Fig. 1b and d) and also inhibited proliferation and migration in VSMCs (Supplement Fig. 5a,b) but not HCAECs (Supplement Fig. 5c,d,e), These data demonstrate that elevated cAMP has divergent effects on proliferation and migration in VSMCs compared to ECs.

### Divergent effects of elevated cAMP on VSMC and EC morphology and actin cytoskeleton remodelling

**Figure 1:**
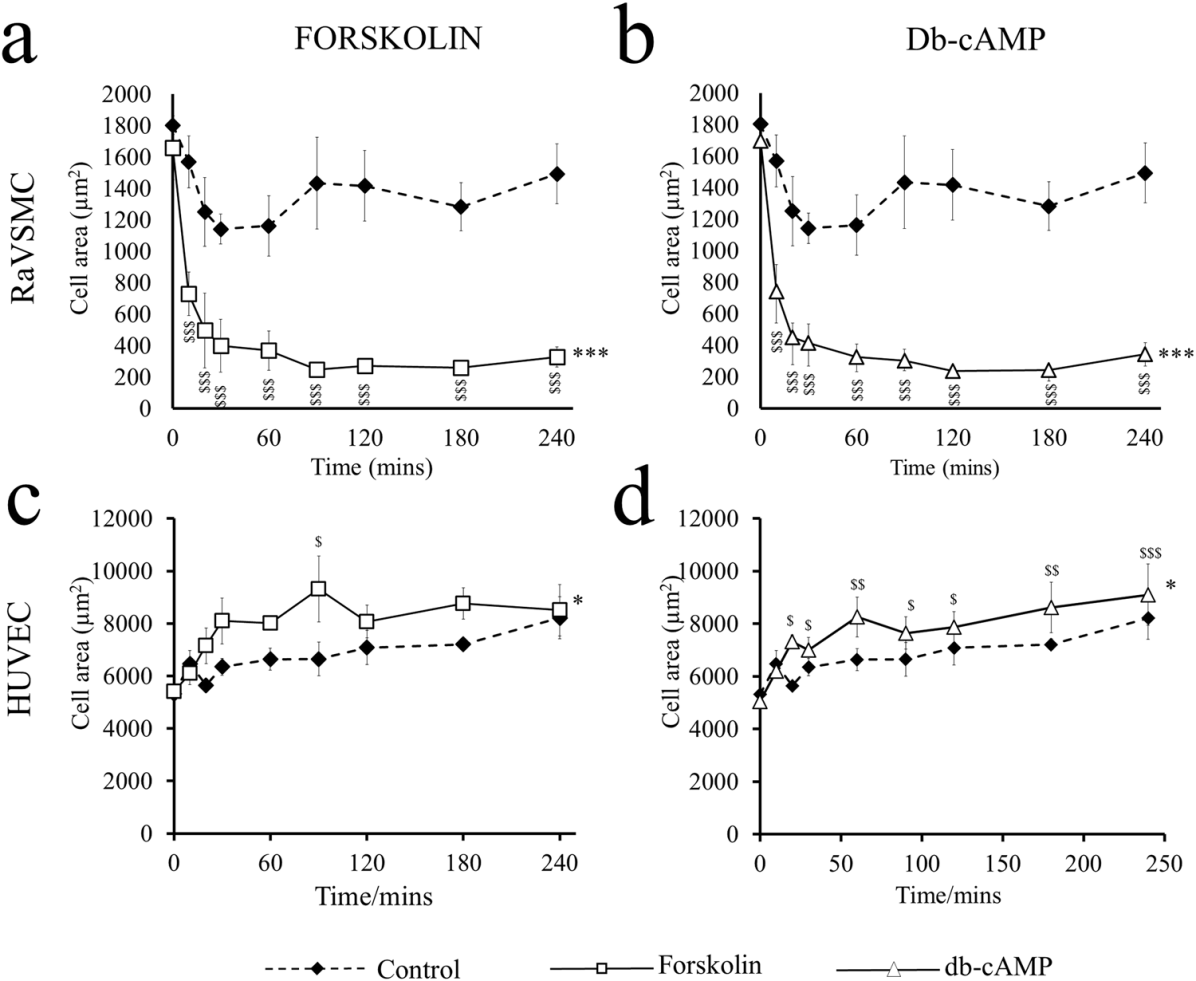
Elevated cAMP inhibits cell spreading in VSMCs but not ECs. RaVSMCs (**a** and **b**; n = 3) and HUVECs (**c** and **d**; n = 3) were serum starved for 18 hours before stimulation with 25 µM forskolin (**a** and **c**) or 500 µM db-cAMP (**b** and **d**) in serum free conditions for the indicated times and total cell area assessed by image analysis of phase contrast images using ImageJ software. Water vehicle control is common to both conditions. *Indicates p < 0.05, ***indicates p < 0.001; Two way repeated measures ANOVA. ^$^Indicates p < 0.05, ^$$^indicates p < 0.01 ^$$$^indicates p < 0.001 vs timepoint zero; One-way ANOVA with student Newman Kuels post test.

To investigate further the functional significance of cAMP-induced cytoskeletal changes on proliferation/migration in VSMCs and HUVECs, we performed detailed time-course experiments. In agreement with previous studies, stimulation of serum starved VSMCs with either forskolin or db-cAMP resulted in induction of a condensed, stellate morphology, characterised by reduced cell spreading (Fig. 1a,b and Supplement Fig. 6). We found that acquisition stellate morphology was evident after only 10 minutes (from 1696 ± 167 µm^2^ in control to 741 ± 160 µm^2^ for db-cAMP, p < 0.001, and to 731 ± 138 µm^2^ for forskolin, p < 0.001) and persisted for at least 240 minutes (Fig. 1a,b). In HUVECs, forskolin or db-cAMP stimulation did not induce stellate morphology (Supplement Fig. 6) but instead resulted in small but significant (p < 0.05; two-way ANOVA) increase in cell spreading (Fig. 1c,d and Supplement Fig. 6).

**Figure 2:**
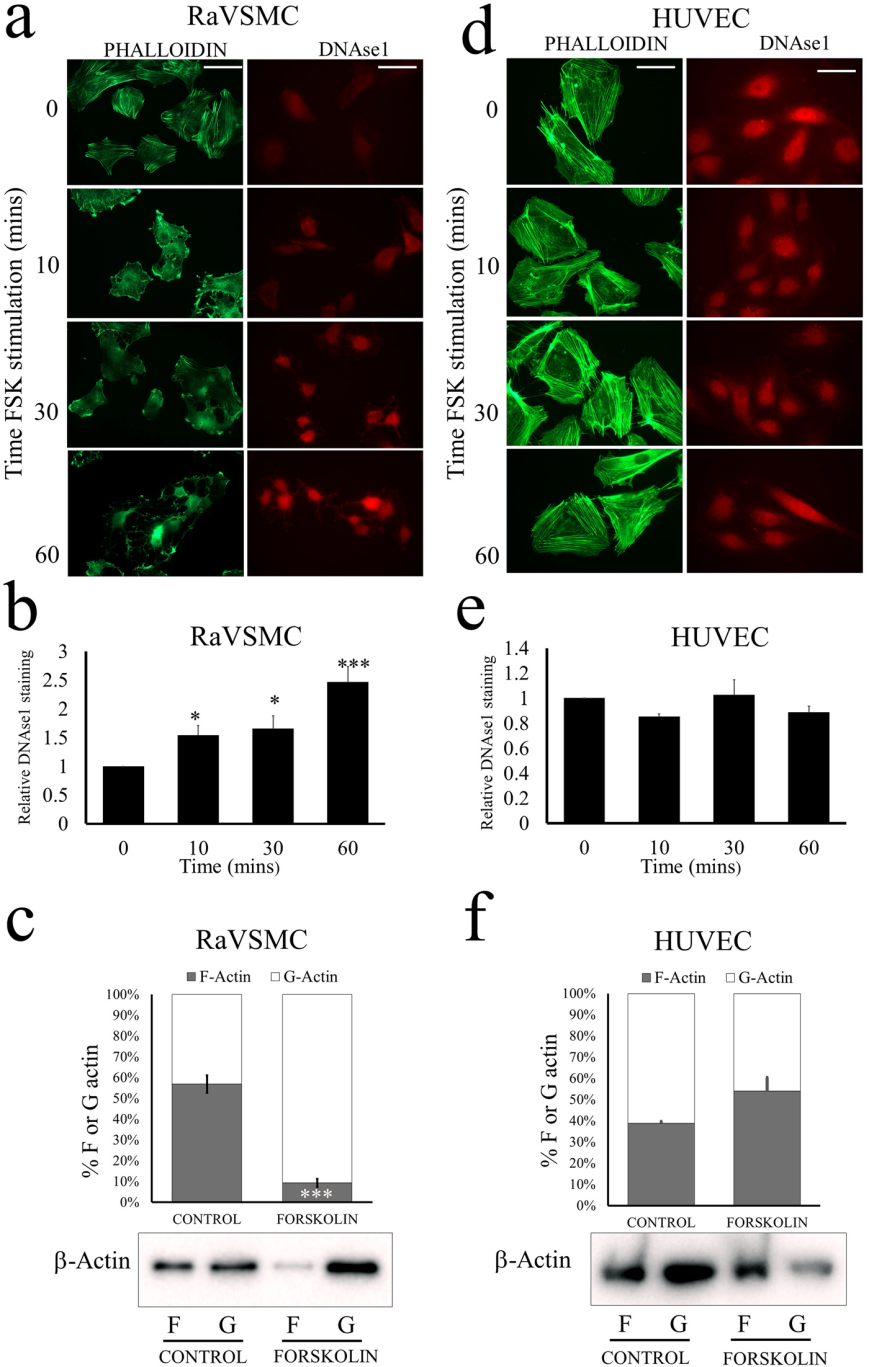
Elevated cAMP inhibits actin polymerisation in VSMCs but not ECs. RaVSMCs (**a**–**c**) and ECs (**d**–**f**) were serum starved for 4 hours before stimulation with 25 µM forskolin (FSK) in serum free conditions for the indicated times. Polymerised actin (F-actin) filaments and actin-monomer (G-actin) were detected using Phalloidin (Green) and DNAse 1 (Red) staining of paraformaldehyde fixed cells. DNAse1 staining of VSMCs (n = 6) and HUVECs (n = 5) was quantified by densitometric analysis (**b** and **e**, respectively). Quantification of F- and G- actin levels by Western blotting (**c** and **f**; cropped blots shown). *Indicates p < 0.05, ***indicates p < 0.001; one-way with replication ANOVA and Student Newman Keuls post-test. Bar indicates 50 µm.

Analysis of F-actin stress fibres in VSMCs using phalloidin staining demonstrated a rapid loss of F-actin within 10 minutes of forskolin stimulation that persisted for at least 60 minutes (Fig. 2a; left panel). Loss of F-actin implies impaired actin-polymerisation and increased actin monomer. To test this, we stained cells with Alexa-fluor-568-conjugated DNAse1, which specifically binds actin monomer^36^. DNAse1 staining in control cells was weak, indicating low levels of actin monomer, but rapidly increased (by 1.54 ± 0.167 fold; p < 0.05 after 10 minutes and by 2.47 ± 0.27 fold; p < 0.001 after 60 minutes of forskolin stimulation; Fig. 2a; right panel and 2B). To further confirm these changes in the actin cytoskeleton, we quantified levels of F- and G-actin using selective solubilisation of G-actin in Triton-X-100, as previously described^37^. This demonstrated that forskolin stimulation of VSMC resulted in a significant reduction in F-actin and a significant increase in G-actin (Fig. 2c).

In HUVECs, no reduction in stress fibres was detected after forskolin stimulation (Fig. 2d; left panels). On the contrary, forskolin stimulation resulted in a more intense staining of cortical actin stress fibres in the cell periphery. No change in DNAse1 stained actin monomer was detected after forskolin stimulation (Fig. 2d; right panels and 2E). Quantification of F- and G-actin levels by selective Triton-X-100 solubilisation also did not detect any significant change in F- or G-actin (Fig. 2f) Taken together, these data demonstrate that elevated cAMP induces rapid loss of F-actin stress fibres, increased actin monomer levels and a condensed stellate morphology in VSMCs but not in ECs.

### Divergent effects of elevated cAMP on SRF-dependent gene expression in VSMCs and ECs

**Figure 3:**
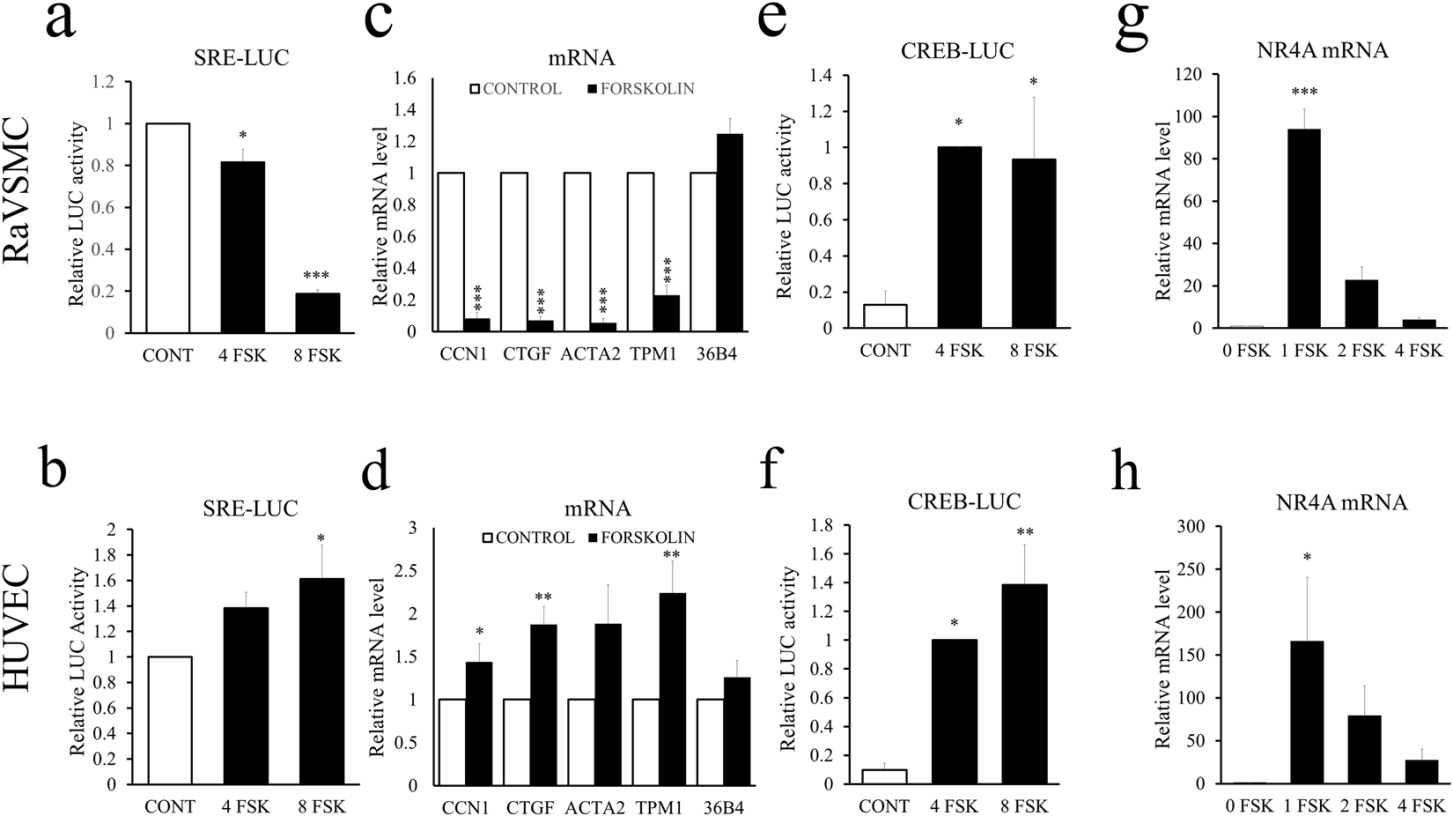
Elevated cAMP inhibits SRF-dependent transcription in VSMCs but not ECs. RaVSMCs (**a**; n = 3, **e**; n = 3) and HUVECs (**b**; n = 4, f; n = 7) were transfected with SRE-LUC (**a**,**b**) or CREB-LUC (**e**,**f**) and stimulated with 25 µM forskolin (FSK) for 4 or 8 hours in the presence of 5% serum and lysates assayed for luciferase activity. RaVSMCs (**c**,**g**) and HUVECs (**d**,**h**) were stimulated with 25 µM forskolin for 4 hours or the indicated times and mRNA levels of indicated genes quantified by RT-qPCR. *Indicates p < 0.05, **indicates p < 0.01, ***indicates p < 0.001 relative to control; one-way with replication ANOVA Student Newman Keuls post-test.

Given that the activity of SRF has been shown to depend on the level of actin-polymerisation in fibroblasts^30, 31^, we compared the activity of SRF in VSMCs and ECs in response to elevated cAMP. Stimulation of VSMCs with forskolin for 4 or 8 hours resulted in significant inhibition (to 0.82 ± 0.06 fold after 4 hours, p < 0.05 and to 0.19 ± 0.06 fold after 8 hours, p < 0.001) of SRE-dependent luciferase reporter gene (SRE-LUC) activity (Fig. 3a). In contrast, SRE-LUC activity was not significantly different after 4 hours and significantly increased after 8 hours (to 1.61 ± 0.28 fold, p < 0.05) of forskolin stimulation in ECs (Fig. 3b). We next used RT-qPCR to quantify effects on endogenous SRF-target genes (CCN1, CTGF, ACTA2 and TPM1). Forskolin stimulation of VSMCs resulted in a significant reduction in mRNA levels of CCN1, CTGF, ACTA2 and TPM1 but not the house-keeping gene 36B4 (Fig. 3c). However, forskolin stimulation of ECs significantly increased mRNA levels of CCN1, CTGF, and TPM1. ACTA2 and 36B4 levels were not significantly different (Fig. 3d). We next quantified effects on CREB-dependent gene expression in order to investigate whether the observed divergent effects on SRF-dependent gene expression were simply due to generalised differences in cAMP responses. Forskolin stimulated CREB-dependent luciferase reporter (CREB-LUC) activity by a similar magnitude (approximately ten fold) and resulted in a rapid transient stimulation of NR4A mRNA, a known CREB-target gene, in both VSMCs and ECs (Fig. 3g and h). These data demonstrate divergent regulation of SRF activity, despite similar regulation of CREB activity by cAMP in VSMCs and ECs.

### Divergent effects of adenosine, Cicaprost and elevated cAMP on MKL1 and MKL2 cytoplasmic-nuclear shuttling in VSMCs and ECs

**Figure 4:**
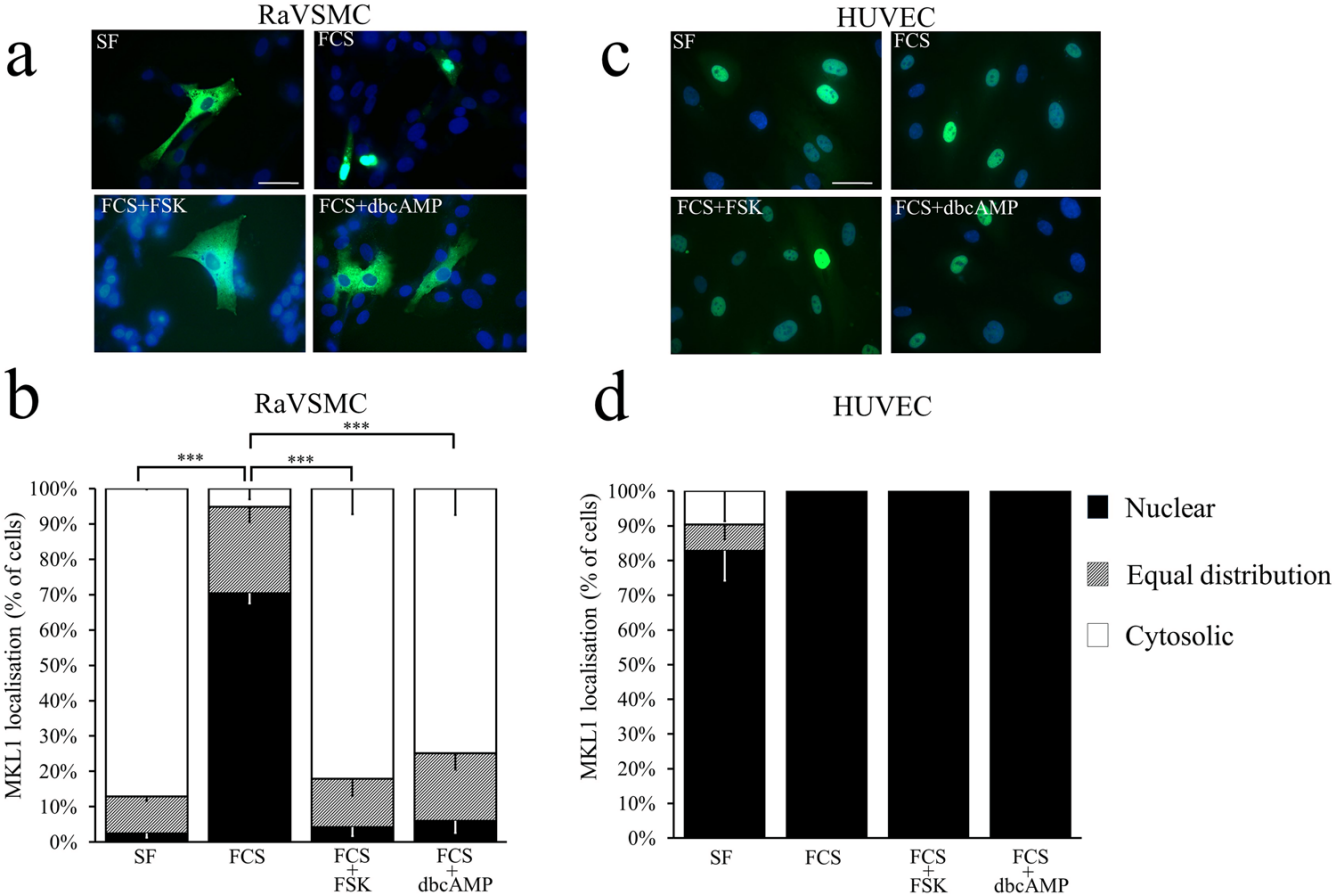
Elevated cAMP inhibits MKL1 nuclear localisation in VSMCs but not ECs. RaVSMCs (**a**,**b**; n = 3) and HUVECs (**c**,**d**; n = 3) were infected with adenoviral vectors expressing GFP-MKL-1. Cells were serum starved for 4 hours before being stimulated for 1 hour with 10% FCS in the presence of either 25 µM forskolin (FSK) or 500 µM db-cAMP, as indicated. Cells were analysed for cellular localisation of MKL1 by fluorescence microscopy (**a** and **c**). Cellular localisation of MKL1 (classified as either nuclear, cytoplasmic or equally distributed between the cytoplasm and nucleus) was quantified by image analysis (**b** and **d**). Images are representative of at least three separate experiments. ***Indicates p < 0.0001 with respect to nuclear localisation; one-way with replication ANOVA and Student Newman Keuls post-test. Bar indicates 50 µm.

We next investigated the mechanisms underlying the divergent regulation of SRF activity by cAMP in VSMCs and ECs. Megakaryoblastic leukemia 1 (MKL1) and Megakaryoblastic leukaemia 2 (MKL2) are both SRF co-factors that contain N-terminal actin-binding RPEL domains, which in MKL1 have been shown to control cytoplasmic:nuclear shuttling, and hence MKL1:SRF activity, in an actin-dependent manner^30^. We therefore studied the regulation of MKL1 and MKL2 cytoplasmic:nuclear shuttling in response to elevated cAMP in VSMCs and ECs. MKL1 and MKL2 protein are expressed in RaVSMCs, HuVSMCs and HUVECs (Supplement Fig. 7). In quiescent VSMCs, MKL1 was predominantly cytoplasmic (only 2.5 ± 1.3% of cells with nuclear MKL1) but rapidly translocated to the nucleus (70.4 ± 2.6% of cells with nuclear MKL1; p < 0.001 vs serum free) within 1 hour of serum stimulation (Fig. 4a and b). Importantly, stimulation with forskolin or db-cAMP completely prevented serum stimulated (Fig. 4) MKL1 nuclear localisation. A similar significant inhibition of serum-induced nuclear localisation of endogenous MKL1 was detected by cell fractionation and western blotting (Supplement Fig. 8). Furthermore, stimulation of VSMCs with BAY60-6583 or Cicaprost also significantly inhibited basal and serum induced nuclear localisation of MKL1 (Supplement Fig. 9).

In HUVECs and HCAECs, MKL1 was predominantly nuclear under serum starvation and remained nuclear after serum stimulation (Fig. 4c and d and Supplement Figs 10 and 11). MKL1 remained nuclear even after prolonged (18 hours) serum starvation (Supplement Fig. 11). Importantly, stimulation with forskolin or db-cAMP had no effect on MKL1 localisation in HUVECs (Fig. 4) or HCAECs (Supplement Fig. 11), with 100% of cells maintaining nuclear-localised MKL1, even after 18 hours of stimulation (Supplement Fig. 11). Elevation of endogenous cAMP with BAY60-6583 or Cicaprost also had no effect of the nuclear localisation of MKL1 in HCAECs (Supplement Fig. 10).

**Figure 5:**
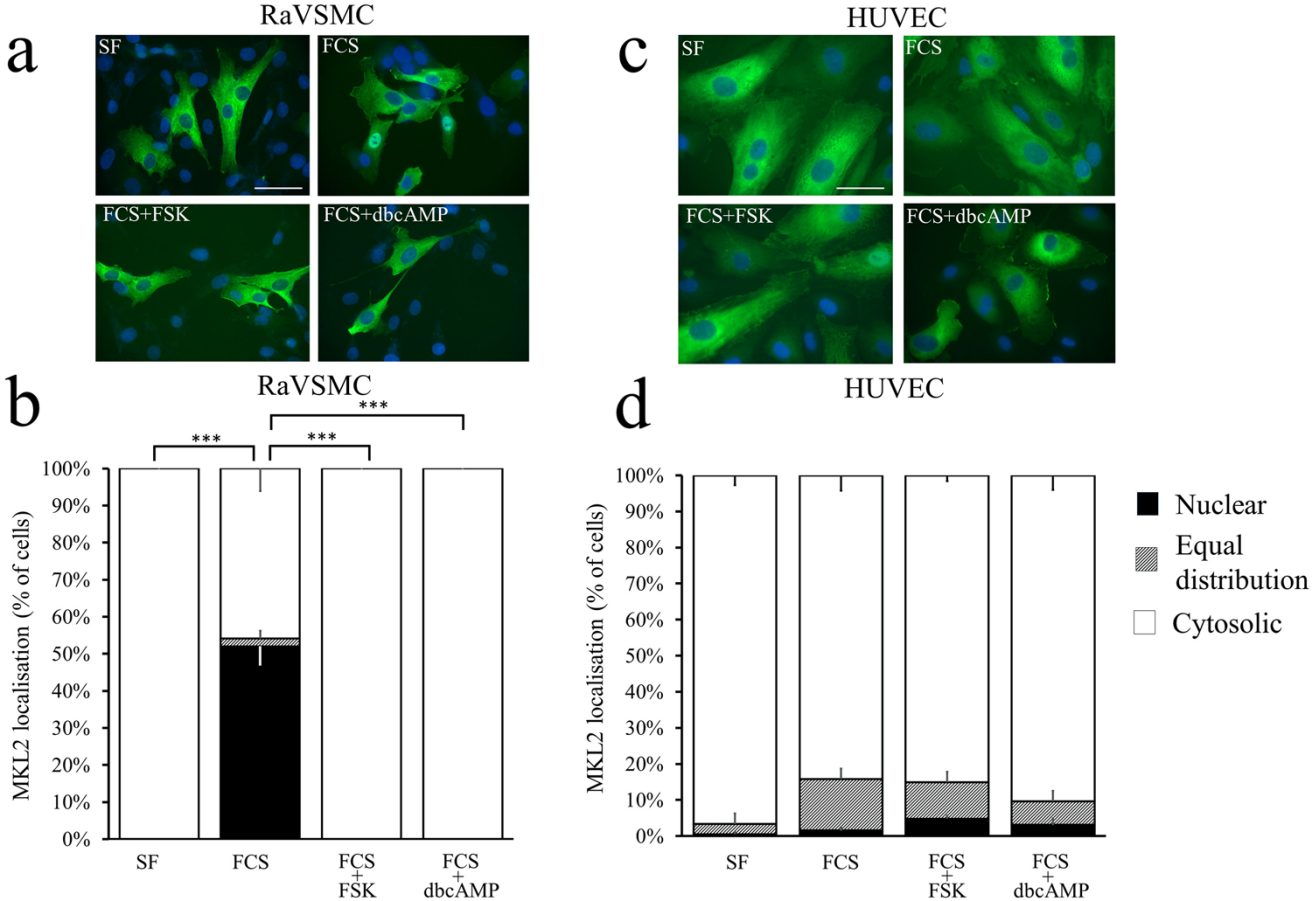
Elevated cAMP inhibits MKL2 nuclear localisation in VSMCs but not ECs RaVSMCs (**a**,**b**; n = 3) and HUVECs (**c**,**d**; n = 3) were infected with adenoviral vectors expressing GFP-MKL-2. Cells were serum starved for 4 hours before being stimulated for 1 hour with 10% FCS in the presence of either 25 µM forskolin (FSK) or 500 µM db-cAMP, as indicated. Cells were analysed for cellular localisation of MKL2 by fluorescence microscopy (**a** and **c**). Cellular localisation of MKL2 (classified as either nuclear, cytoplasmic or equally distributed between the cytoplasm and nucleus) was quantified by image analysis (**b** and **d**). Images are representative of at least three separate experiments. ***Indicates p < 0.0001 with respect to nuclear localisation; One-way with replication ANOVA and Student Newman Keuls post-test. Bar indicates 50 µm.

In quiescent VSMCs, MKL2 was exclusively cytoplasmic but was rapidly translocated to the nucleus within 1 hour of serum stimulation (Fig. 5a and b). Stimulation with forskolin or db-cAMP completely suppressed the serum stimulated (Fig. 5a and b) nuclear localisation of MKL2. BAY60-6583 and Cicaprost also significantly inhibited serum stimulated and basal MKL2 nuclear localisation (Supplement Fig. 9d–f). In HUVECs, MKL2 exhibited a constitutively-cytoplasmic localisation that was not significantly modulated by serum stimulation, with or without forskolin or db-cAMP co-stimulation (Fig. 5c and d).

### Divergent roles of RhoA-actin signalling in cAMP-dependent regulation of MKL1 and MKL2 in VSMCs

**Figure 6:**
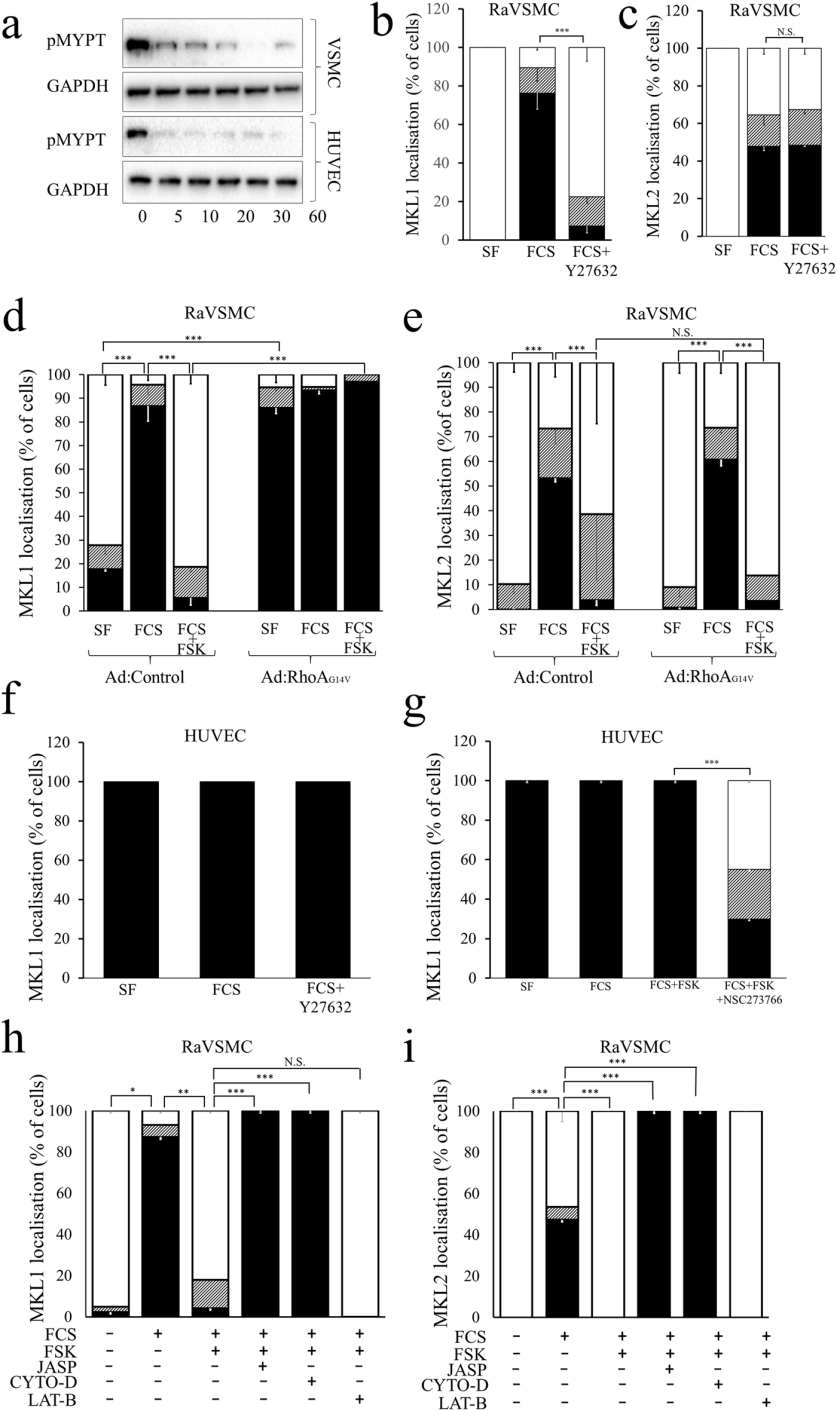
Regulation of MKL-1 and -2 by RhoA and actin monomer in VSMCs. RaVSMCs and HUVECs were treated with 25 µM forskolin for indicated times and total cell lysates analysed for phosphorylated MYPT by western blotting (**a**; n = 1; cropped blots shown). RaVSMCs were serum starved for 4 hours and stimulated for 1 hour with 10% FCS ± 10 µM Y27632 and cellular localisation of MKL1 (**b**; n = 3) or MKL2 (**c**; n = 3) quantified (**b**; n = 3). RaVSMCs were infected with adenoviral vector expressing GFP-MKL1 (**d**; n = 3) or GFP-MKL2 (**e**; n = 3) together with either control virus (Ad:control) or virus expressing constitutively-active RhoA (Ad:RhoA_G14V_). Cells were serum starved for 4 hours before being stimulated for 1 hour with 10% FCS in the presence or absence of 25 µM forskolin and MKL-1 and -2 localisation quantified. HUVECs were serum starved for 4 hours before a 1 hour stimulation with 10% FCS ± 10 µM Y27632 and cellular localisation of MKL1 quantified (**f**; n = 3). MKL1 localisation in HUVECs that were serum starved for 4 hours before a 1 hour stimulation with 10% FCS in the presence of 25 µM forskolin (FSK) and/or 10 µM NSC273766 (**g**; n = 3). RaVSMCs expressing GFP-MKL1 (**h**; n = 3) or GFP-MKL2 (**i**; n = 3) were serum starved for 4 hours before being stimulated for 1 hour with 10% FCS in the presence or absence of 25 µM forskolin and 1 µM Jasplakinolide, 2 µM Cytochalsin-D (CYTO-D) or 5 µg/ml latrunculin B (LAT-B), as indicated. Black bars indicate nuclear localisation, white bars cytosolic localisation and striped bars mixed localisation of MKL1/2. *Indicates p < 0.05, **indicates p < 0.01, ***indicates p < 0.001; One-way with replication ANOVA and Student Newman Keuls post-test.

Our data demonstrated that cAMP-dependent reduction in actin-polymerisation and increased levels of actin monomer is associated with inhibition of MKL1 and MKL2 cytoplasmic:nuclear shuttling in VSMCs. Given that elevated cAMP has been linked to inhibition of RhoA-ROCK signalling, we tested the role of RhoA-ROCK and actin monomer in the regulation of MKL1 and MKL2 by cAMP. We initially asked if cAMP differentially modulates RhoA-ROCK signalling in VSMCs and ECs. Treatment of VSMCs and HUVECs with forskolin rapidly inhibited phosphorylation of the ROCK substrate, MYPT (Fig. 6a), indicating inhibition of RhoA-ROCK signalling in both cell types. Inhibition of RhoA-ROCK signalling using Y27632 significantly reversed serum induced nuclear translocation of MKL1 but not MKL2 in VSMC (Fig. 6b and c) suggesting that MKL1 is RhoA-ROCK-dependent and MKL2 is RhoA-ROCK independent in VSMC. Consistent with this, expression of a constitutively-active RhoA mutant (Ad:RhoA_G14V_) forced nuclear localisation of MKL1, which could not be overcome by forskolin (Fig. 6d). In contrast, the nuclear translocation of MKL2 and its inhibition by forskolin in VSMCs was not modulated by expression of constitutively-active RhoA (Fig. 6e) suggesting that nuclear exclusion of MKL2 by cAMP occurs independently of RhoA inhibition.

In HUVECs, Y27632 had no effect on the constitutively nuclear MKL1 (Fig. 6f) suggesting that MKL1 is RhoA-ROCK-independent in EC. We therefore tested the role of Rac1. Co-stimulation of HUVECs with forskolin and the Rac1 inhibitor NSC273766 resulted in a significant reduction in nuclear MKL1 (Fig. 6g).

We next tested the role of increased actin-monomer (G-actin) in the cAMP-dependent regulation of MKL1 and MKL2 in VSMCs. We depleted actin monomer levels using jasplakinolide, which promotes actin polymerisation, or cytochalasin-D, which sequesters actin monomers. Conversely, latrunculin-B, which inhibits actin polymerisation was used to elevate actin monomer. Latrunculin –B significantly reduced levels of F-actin and increased G-Actin, while Jasplakinolide significantly increased levels of F-actin and reduced G-Actin (Supplement Fig. 12). Co-stimulation with jasplakinolide or cytochalasin-D completely reversed the inhibitory effects of forskolin on MKL1 (Fig. 6h) and MKL2 (Fig. 6i) nuclear localisation. Stimulation with latrunculin-B induced an exclusively cytoplasmic localisation of both proteins. Taken together, these data indicate that cAMP-regulation of both MKL1 and MKL2 is dependent on elevation of actin-monomer.

### Regulation of proliferation and migration by MKL1 and MKL2 in VSMCs and ECs

**Figure 7:**
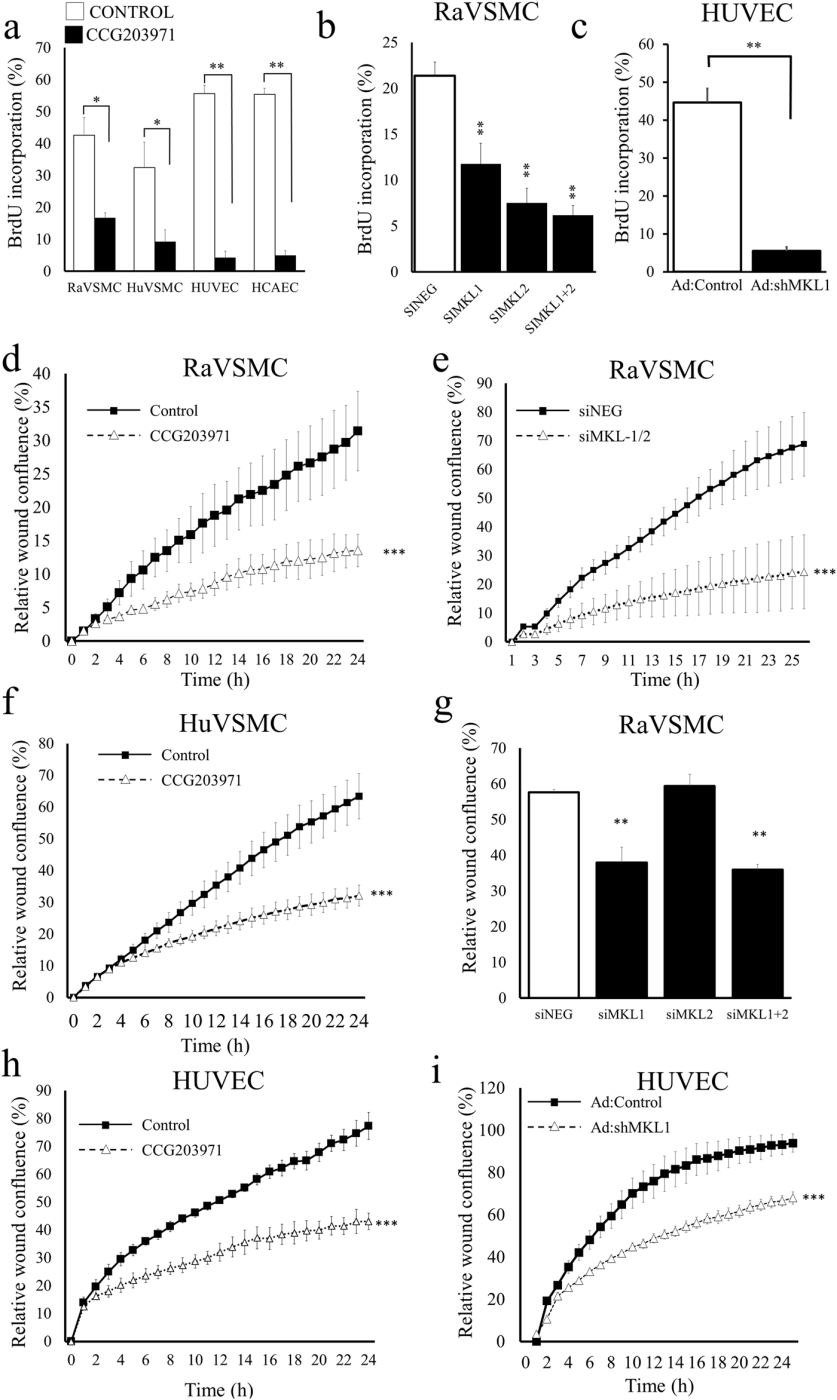
Role of MKL-1 and -2 in VSMC and EC proliferation and migration. Asynchronously proliferating RaVSMCs, HuVSMCs and HUVECs were treated with 20 µM CCG203971 for 24 hours. BrDU was included for the last 6 hours to label proliferating cells (**a**; n = 3). RaVSMCs were transfected with siRNA targeting MKL1, MKL2 or MKL1 + MKL2 and cells labelled with BrDu 48 hours after transfection (**b**; n = 3). HUVECs were infected with adenovirus expressing shRNA targeting MKL1 (Ad:shMKL1) and cells labelled with BrDu 48 hours after infection (**c**; n = 3). Migration of RaVSMCs (**d**,**e**,**g**; n = 3), HuVSMCs (**f**; n = 3) and HUVECs (**h**,**i**; n = 3) in presence of 20 µM CCG203971 (**c**,**e**,**f**), transfected with siRNA targeting MKL1, MKL2 or MKL1 + MKL2 (**h**,**i**) or infected with Ad:shMKL1 (**j**) was analysed using IncuCyte real-time scratch wound assays. *Indicates p < 0.05, **indicates p < 0.01, ***indicates. One-way with replication ANOVA and Student Newman Keuls post-test (**a**,**b**,**g**). Student’s T-test (**c**). Two-way ANOVA (**d**,**e**,**f**,**h**,**i**).

Our data demonstrates that the anti-mitogenic and anti-migratory properties of cAMP in VSMCs are associated with inhibition of MKL1 and MKL2 nuclear localisation. In ECs, by contrast, cAMP does not inhibit MKL1 nuclear localisation and does not inhibit proliferation or migration. The implication is that inhibition of MKL1 and/or MKL2 nuclear localisation may underlie the anti-mitogenic and anti-migratory properties of cAMP in VSMCs. To test this further, we analysed the function of MKL1 and MKL2 in VSMCs and ECs using pharmacological inhibition and gene-silencing. Treatment of cells with the second-generation MKL-inhibitor, CCG203971, resulted in a significant inhibition of cell proliferation, measured by BrdU incorporation in human and rat VSMCs, HUVECs and HCAECs (Fig. 7a). We validated the effects of CCG203971 using MKL1 and MKL2 silencing. Transient transfection of VSMC with MKL1/2 siRNA reduced MKL1 and MKL2 protein, without affecting GAPDH (Supplement Fig. 13a). Adenoviral-mediated expression of MKL1 shRNA in HUVECs also reduced MKL1 protein without affecting GAPDH (Supplement Fig. 13b). Silencing of MKL1, MKL2 or simultaneous silencing of both in VSMCs also significantly inhibited VSMC proliferation (Fig. 7b). In ECs, silencing of MKL1 alone (since this was the only MKL-factor displaying nuclear localisation in these cells) also significantly inhibited proliferation (Fig. 7c). Treatment with CCG203971 also significantly inhibited migration of VSMCs (Fig. 7d and e), HUVECs (Fig. 7f) and HCAECs (Supplement Fig. 14). Dual silencing of MKL1 plus MKL2 or individual silencing of MKL1, but not MKL2 significantly inhibited VSMC migration (Fig. 7g and h). In ECs, MKL1 silencing significantly inhibited endothelial migration (Fig. 7i). Hence, although cAMP elevation differentially affected MKL1 nuclear translocation in VSMCs and ECs, the downstream effects of MKL1 appeared to be similar.

## Discussion

In this study we investigated the regulation of the SRF co-factors, MKL1 and MKL2, by cAMP signalling in VSMCs and ECs and the role of this mechanism in regulation of cell proliferation and migration. We demonstrate that cAMP-induced actin-depolymerisation prevents mitogen-induced nuclear localisation of MKL1 and 2 in VSMCs and that this mechanism underlies, at least in part, the anti-mitogenic and anti-migratory effects of cAMP in these cells. In detail, cAMP elevation in VSMCs using either forskolin, cAMP-analogues or physiological GPCR agonists rapidly inhibited RhoA-ROCK signalling, inhibited actin polymerisation (F-actin), increased actin-monomer (G-actin) levels and completely prevented mitogen-induced nuclear localisation of both MKL1 and MKL2. Furthermore, we show that the divergent effects of cAMP on VSMC and EC proliferation and migration can be explained by differences in actin-remodelling and MKL1 and 2 cytoplasmic:nuclear shuttling. Although RhoA-ROCK signalling was inhibited in both cell types, stimulation of cAMP signalling in venous or arterial ECs did not inhibit actin polymerisation, elevate actin-monomer levels or induce exclusion of MKL1 from the nucleus. MKL2 was constitutively cytoplasmic in these cells. As a result, proliferation and migration of ECs was not inhibited in response to elevated cAMP or GPCR activation. In some cases, cAMP elevation actually stimulated EC proliferation and migration (Supplement Figs 1–3)^38^. Although these stimulatory effects were modest, they contrasted with the large inhibitory effects observed in VSMCs Previous studies have shown that MKL1 expression is low in healthy arteries but is elevated after wire-injury to femoral arteries in mice^35^. Mice deficient in MKL1 develop smaller injury-induced intimal lesions than their wild type counterparts and display attenuated atherosclerosis, suggesting that MKL1 plays a central role in vascular remodelling^35^. Although little is known about the function of MKL2 in the vascular cells, MKL2-deficient mice die in late gestation of vascular defects^39^. We now show using pharmacological and siRNA-mediated silencing that MKL1 is essential for VSMC and EC proliferation and migration. MKL2 also plays an important role in controlling VSMC proliferation but not migration. Our new data highlights the divergent effects of cAMP on actin remodelling and hence MKL1/2 regulation between VSMCs and ECs and helps explain the opposite effects of cAMP on proliferation and migration in these cells.

Precisely how cAMP-induced actin dynamics modulates VSMC behaviour has remained poorly understood but likely involves actin-sensitive transcription factors. SRF activity is sensitive to RhoA-actin dynamics and plays a central role in controlling VSMC differentiation as well as promoting expression immediate-early genes required for cell proliferation and migration, such as c-fos^40^. For example, activated RhoA mutants that promote actin-polymerisation induce the expression of SRF-dependent reporter genes while inhibition of RhoA with C3 transferase blocks their induction by mitogenic stimuli^41^. This has led to the model where free G-actin inhibits SRF activity and this inhibition is relieved by Rho GTPase- mediated actin polymerisation. Alterations in actin dynamics appear to be necessary for the regulation of SRF by a wide range of extracellular signals, including growth factors, lysophophatydic acid, extracellular matrix and GPCR agonists^31, 42^. Treatment of cells with actin polymerisation inhibitors or actin polymerisation agents demonstrated that SRF activity is responsive to changes in the G-actin pool^31^. We previously demonstrated the cAMP inhibited members of the Rho GTPases, including RhoA and Rac1 in VSMCs^19^ and here we show that elevated cAMP rapidly induces loss of F-actin and an increase in G-actin in VSMCs but not ECs. This is associated with inhibition of SRF-dependent transcription in VSMCs but not ECs. In VSMCs, it is likely that reduced actin-polymerisation and the concomitant increase in G-actin is responsible for repression of SRF activity. Interestingly, in ECs, SRF-dependent transcription is actually stimulated by elevated cAMP. The mechanism is not clear but we observed an increase in phalloidin stained cortical F-actin stress fibres after forskolin stimulation; although we could not detect any reduction in global G-actin. However, we cannot exclude the possibility that small undetectable changes in the levels or localisation of G-actin may be responsible for the forskolin-mediated increase in SRF activity in these cells. Nevertheless, our data clearly demonstrates that activation of cAMP-sensitive signalling pathways has divergent effects on actin-dynamics and SRF activity in VSMCs and ECs and we suggest that this is responsible, at least in part, for the opposing effects of cAMP on the proliferation and migration of these cells.

Why cAMP has such different effects on actin dynamics and SRF activity in VSMCs and ECs is currently unclear but is likely to reflect differences is the regulation of Rho GTPases in these cells. For example, we and others previously demonstrated inhibition of RhoA and Rac1 in VSMCs in response to elevated cAMP signalling^19^. A similar inhibition of RhoA has been reported in ECs, and has been implicated in increased EC spreading and reduced EC barrier permeability in response to cAMP^43^. We detected a rapid inhibition of RhoA-ROCK signalling in both VSMCs and ECs in response to elevated cAMP. However, nuclear localisation of MKL1 in VSMCs was ROCK-dependent but in ECs it was ROCK-independent. Our data suggests that in ECs, cortical actin and nuclear MKL1 is maintained by a RhoA-ROCK independent mechanism. Our data supports a role for Rac1 in maintaining nuclear MKL1 in ECs. In ECs, cAMP induces Rac1 activity^44, 45^, which is associated with increase cortical actin polymerisation. Inhibition of Rac1 using NSC273766 in the presence of forskolin reduces nuclear MKL1 levels, suggesting that Rac1-mediated cortical actin polymerisation is responsible for maintaining nuclear MKL1 in ECs and may, at least in part, account for our observed differences in MKL translocation between VSMCs and ECs.

MKL1 and MKL2 sense G-actin concentrations using their N-terminal RPEL domains and transduce signals to SRF^30, 46, 47^. Stimuli that inhibit Rho GTPases or actin polymerisation typically sequester MKL1 and 2 in the cytoplasm whereas factors that activate Rho GTPases or promote actin polymerisation induce their nuclear translocation and hence SRF activation. Here we show that elevated cAMP completely blocks mitogen induced nuclear localisation of MKL1 and MKL2 in VSMCs. In the case of MKL1, this is likely mediated by the inhibitory effects of cAMP on RhoA, as forced expression of a constitutively-activated RhoA completely prevented the inhibitory effects of cAMP on MKL1 nuclear localisation. Although mitogens induced nuclear localisation of MKL2 in VSMCs and this was also blocked by cAMP, this effect was not reversed by activated RhoA, suggesting the involvement of different mechanisms controlling MKL1 and MKL2 cytoplasmic:nuclear shuttling in VSMCs. Similar differences in the regulation of MKL1 and MKL2 have been reported previously. For example, mechanical force activates RhoA and induces nuclear translocation of MKL1 but not MKL2 in myofibroblasts, whereas both respond in a similar way to serum stimulation in these cells^48^. The mechanisms underlying the differences in MKL1 and MKL2 regulation are currently unclear and certainly warrant further research. This may reflect subtle differences in their regulation by different Rho GTPases or a requirement for additional factors for MKL1/2 nuclear import, such as the previously reported role of serine-454 phosphorylation^49^. Interestingly, our data indicates that cAMP controls both MKL1 and MKL2 by elevating G-actin in VSMCs. Depletion of G-actin completely reversed the effect of cAMP on both proteins, suggesting that regulatory differences lie upstream of G-actin. In ECs, MKL2 also displayed a different pattern of regulation to MKL1. In these cells, MKL1 was constitutively nuclear whereas MKL2 was constitutively cytoplasmic. Again, this may reflect a requirement for additional mechanisms for MKL2 nuclear localisation that are not active in ECs. Moreover, MKL1 and MKL2 were unresponsive to serum mitogens or cAMP elevation in ECs, remaining constitutively nuclear or cytoplasmic respectively. The constitutively nuclear MKL1 likely reflects the fact that G-actin levels were unaffected by cAMP in these cells and as discussed above, is this probably due to activation of Rac1-mediated cortical actin polymerisation.

Several lines of evidence suggest that MKL1 and MKL2 are involved in controlling proliferation and migration of other cells. For example, dual silencing of MKL1 and MKL2 in NIH3T3 cells suggested an important and complex role in maintaining cell cycle progression and genomic stability^50^. Furthermore, activation of MKL1 using the small molecule isoxazole promoted wound closure *in vivo* in mice^51^. MKL1 and MKL2 are constitutively localized to the nucleus in hepatocellular and mammary carcinoma cells and their depletion suppresses migration and proliferation and anchorage-independent cell growth^52^. Double conditional deletion of MKL1 and MKL2 in the epicardium in mice reduces epicardial cell migration^53^. In addition, the first generation RPEL domain inhibitor, CCG1423, effectively blocks intima formation in wire injured femoral arteries in mice^35^. However, depletion of MKLs in some cell types does not affect proliferation, suggesting cell-type specific effects of these co-factors^54^. Our data obtained using siRNA-mediated silencing and pharmacological inhibition now shows that MKL1 plays a role in promoting proliferation and migration of both VSMCs and ECs, emphasising the similar effects of signalling downstream of MKLs. Our study is the first to provide evidence that MKL2 also plays a key role in controlling VSMC proliferation. Interestingly, depletion of MKL2 in VSMCs had no effect of cell migration despite its clear effects on S-phase entry, suggesting functionally divergent roles of MKL1 and MKL2 in VSMCs. In summary, our data indicates that cell-type specific effects on actin-cytoskeleton dynamics and subsequent nuclear translocation of MKLs explains, at least in part, the divergent properties of cAMP in VSMCs and ECs.

Taken together, our data help to explain the divergent effects of cAMP signalling on VSMC and EC proliferation and migration. Inhibition of actin polymerisation and MKL-dependent SRF activity underlies, at least in part, the inhibitory effects of cAMP on the growth and migration of VSMCs. In ECs, cAMP stimulates increased cortical actin polymerisation, which maintains low levels of G-actin, permits SRF activation and increases EC proliferation and migration. Furthermore, our data showing that MKL2 is inactive (i.e. constitutively cytoplasmic) in ECs but is required for VSMC proliferation suggest that MKL2 may represent a valuable target for future therapies designed to selectively inhibit VSMC proliferation.

## Material and Methods

### Materials

All chemicals were obtained from Sigma unless otherwise stated. Antibodies to MKL1 (#14760) and Lamin A/C (#4777) were from Cell Signalling Technologies. Antibody to GAPDH (MAB374) was from Millipore. Anti-BrdU antibody (B2531) was from Sigma Aldrich.

### Smooth muscle and endothelial cell culture

Male Sprague Dawley rats were killed by cervical dislocation in accordance with schedule 1 of the U.K. Animals (Scientific Procedures) Act 1986 and Directive 2010/63/EU of the European Parliament and with the approval of the University of Bristol. Methods used to culture VSMCs and ECs are described in detail in the Supplement. Human saphenous vein VSMCs (HuVSMCs) at passage 2–8 were generated as described previously^55^ from spare sections of human saphenous vein obtained with informed consent in all cases from patients undergoing coronary artery bypass surgery at the Bristol Royal Infirmary. All procedures were carried out in accordance with the ethical approval (Research Ethics Committee #04/Q2007/6) and the approval of the University of Bristol ethical committee. Approximately ten different batches of rat aortic VSMCs (RaVSMCs), six batches of human saphenous vein VSMCs (HuVSMCs), six batches of human umbilical vein ECs (HUVECs) and three batches of human coronary artery ECs (HCAECs) were used in these studies. All experiments were performed using different batches of cells that were prepared from different animals/donors.

### Real-time scratch wound migration assays

Real-time analysis of migration was performed using a IncuCyte® ZOOM live cell imaging system (Essen BioScience) according to the manufacturer’s instructions. Briefly, cells were seeded (2 × 10^4^ cells/well for RaVSMCs, 1 × 10^4^ cells/well for HuVSMCs and 2 × 10^4^ cells/well for HUVECs) into ImageLock-96 well plates. Wells were scratched using a WoundMaker® tool and phase contrast images of cell migration into the wounded area acquired hourly for 24 hours. Relative wound confluence was calculated using the Cell Migration Image analysis module of the IncuCyte® ZOOM software.

### Quantitative RT-PCR and Western Blotting

Quantification of mRNA and protein levels was performed by RT-qPCR and western blotting respectively, as described previously^56^. Total RNA, extracted using Ambion Pure-Link kits (Thermo Fisher) and was reverse transcribed using QuantiTect RT kit (Qiagen) and random primers. Quantitative PCR was performed using Roche SYBR Green using a Qiagen Roto-Gene Q PCR machine (20′@95 °C; 20′@62 °C; 20′@72 °C). Primers sequences are described in Supplement Table 1. Data were normalised to total RNA. Western blots were performed using a Mini-Protean II system. Proteins were transferred to PVDF membrane using a semi-dry Turbo blotter (Bio-Rad) and detected using ECL and a digital ChemiDoc imaging system (Bio-Rad).

### Plasmids, siRNA and Adenoviral Vectors

Details of all plasmid and viral vectors are described in the Supplement. Silencer Select siRNAs were purchased from Invitrogen and are also described in the Supplement.

### Transient transfection

Plasmid transfection was performed by electroporation using an Amaxa Nucleofector-1.5. 1 × 10^6^ VSMCs were transfected with 3–5 μg of DNA or 100 pmoles of siRNA using the standard Nucleofector program A033.

### Reporter gene luciferase assays

Cells were transfected by electroporation with the indicated promoter reporter plasmids together with pTk-Renilla for normalisation. Cells were stimulated with the indicated agents 24 hours followed by lysis in Promega cell culture lysis buffer. Luciferase and Renilla activity were quantified using the dual reporter assay kit (Promega) according to the manufactures instructions using Glomax luminometer (Promega).

### F:G actin ratio assays

F- and G-actin were separated by triton solubility essentially as previously^37^ described with slight modifications. Following treatment, G-actin was extracted in G-actin extraction buffer (PBS, 10% glycerol, 0.1% triton X-100, 1 mM ATP and complete protease inhibitor) and incubated for 5 min at room temperature with slight agitation. Samples were centrifuged at 15,000 *g* at 4 °C for 5 min. The supernatant (soluble G-actin) was collected. Titon-X-100 insoluble material (F-actin) remaining in the wells and pelleted from the soluble fraction was lysed in reducing Laemmli SDS sample buffer. Samples were analysed by western blotting using a β-actin specific antibody (Sigma).

### cAMP quantification

Intracellular cAMP levels were quantified using the cAMP direct ELISA assay (Abcam) according to the manufacturers instructions.

### Statistical Analysis

After testing for Gaussian distribution, statistical analysis was performed using two-way ANOVA, one-way ANOVA with Student-Newman-Keuls post-test or where appropriate a paired student’s t-test, as indicated. *Indicates p < 0.05, **indicates p < 0.01, ***indicates p < 0.001.

### Data Availability

The datasets generated during and/or analysed during the current study are available from the corresponding author on reasonable request.

## Electronic supplementary material

supplementary Info
