# Supplementary material for: Divergent Regulation of Actin Dynamics and Megakaryoblastic Leukemia-1 and -2 (Mkl1/2) by cAMP in Endothelial and Smooth Muscle Cells
Source: Sci Rep. 2017 Jun 16;7:3681. doi: 10.1038/s41598-017-03337-0 (PMC5473867; doi:10.1038/s41598-017-03337-0)

DIVERGENT REGULATION OF ACTIN DYNAMICS AND MEGAKARYOBLASTIC LEUKEMIA-1 AND -2 (MKL1/2) BY cAMP IN ENDOTHELIAL AND SMOOTH MUSCLE CELLS

SUPPLEMENTARY MATERIAL

Madeleine C Smith[1], Claire Hudson[1], Tomomi E Kimura[1], Stephen J White[2], Graciela B Sala-Newby[1], Andrew C Newby[1] and Mark Bond[1]

[1]School of Clinical Sciences, University of Bristol, Research Floor Level 7, Bristol Royal Infirmary, Bristol BS2 8HW

[2] Manchester Metropolitan University, John Dalton Building, Chester Street, Manchester M1 5GD

Corresponding Author: Dr Mark Bond, Level 7 Queens Building, Bristol Royal Infirmary,

University of Bristol, Bristol, U.K. BS2 8HW. Tel: +44 (0)117 3423586

Email: mark.bond@bris.ac.uk.

SUPPLEMENTARY METHODS

Materials: Forskolin, db-cAMP and CCG203971 were purchased from Sigma Aldrich. BAY60-6583 was purchased from Tocris Bioscience. Cicaprost was purchased from Caymen Chemical.

Smooth muscle and endothelial cell culture: Mixed donor HUVECs were purchased from Promocell and cultured in endothelial cell growth medium (supplemented with 2% FCS, 0.4 % bovine hypothalamic extract, 0.1 ng/ml EGF, 1 ng/ml bFGF, 90 µg/ml heparin and 1 µg/ml hydrocortisone). Three different batches of human coronary artery endothelial cells (HCAECs) were purchased from Promocell and cultured in endothelial growth medium MV2 supplemented with 5% FCS, 5 ng/ml EGF, 10 ng/ml bFGF, 20 ng/ml IGF, 0.5 ng/ml $VEGF_{165}$ and 0.2 ug/ml hydrocortisone. Stimulations were performed in 5% foetal calf serum/DMEM unless otherwise stated. Cells were serum starved for 18 hours or 4 hours, as indicated in figure legends. Proliferation was measured by culture in the presence of 10 µM bromodeoxyuridine (BrdU) for 6 hours. Following fixation in 70% ethanol, incorporated BrdU was detected by immune-histochemical staining as previously described [34]. Typically, all cells (at least 200) in five to ten fields of view were manually counted using ImageJ software. For nuclear and cytosolic fractionation, cells were lysed in hypotonic lysis buffer (10 mM HEPES pH7.4, 10 mM KCl, 1.5 mM $MgCl_2$, 0.05% NP-40, 1 mM PMSF, 1 µg/ml aprotinin, 1µg/ml leupeptin) with homogenisation. Nuclei were pelleted at 1000g and washed in lysis buffer. Nuclear proteins were extracted in SDS lysis buffer (1% SDS, 10 mM Tris pH6.8, 10% glycerol).

Plasmids, siRNA and Adenoviral Vectors: PathDetect SRE reporter plasmid (SRE-LUC) was purchased from Agilent Technologies. CREB reporter plasmid (CREB-LUC; α-168) was a gift from Prof Stan McKnight [35]. Adenoviruses expressing GFP-MKL1 and GFP-MKL2 were generated by sub-cloning the coding sequence from pEGFPN-MKL1 and pEGFPN-MKL2 plasmids to pDC515 (Microbix). Viruses were generated from the resulting pDC515 vectors as previously described [34]. Adenovirus expressing constitutively-active $RhoA_{G14V}$ has been described previously [34]. Adenovirus expressing short-hairpin shRNA targeting human MKL1, hairpin sequences were designed using the prediction tool avaliable at http://cancan.cshl.edu/RNAi_central/RNAi.cgi?type=shRNA. Sequences used to silence MKL1 were 5'-TGC TGT TGA CAG TGA GCG CGC CGA TGA CCT CAA TGA GAA GTA GTG AAG CCA CAG ATG TAC TTC TCA TTG AGG TCA TCG GCT TGC CTA

CTG CCT CG- 3'. shRNA targeting and firefly luciferase (5'-CGC CTG AAG TCT CTG ATT AAT AGT GAA GCC ACA GAT GTA TTA ATC AGA GAC TTC AGG CGG T-3') was used as a control. shRNA sequences were embedded in the backbone of the primary microRNA-30, as described previously [36]. DNA sequences for modified microRNA-30 were synthesized by Genscript, cloned into the Nhe1-BamH1 sites of the shuttle vector pDC515 and adenoviruses generated as described above. Silencer Select siRNAs targeting rat *MKL1* (s163756) and rat *MKL2* (ABX00O2) were purchased from Life Technologies. Control siRNA was Silencer Select negative control (#4390844).

F and G actin staining and quantification: For detection of F- or G-actin cells were cultured on sterile glass 13mm diameter coverslips. F-actin stress fibres were detected by staining with Alexa-fluor-488 labelled phalloidin (Life Technologies). Briefly, cells were fixed in 4% paraformaldehyde for 10 minutes at room temperature before being permeabilised in 0.1% Triton-X-100 in PBS for 5 minutes. Cells were then stained with phalloidin for 30 minutes in accordance with the manufacturer's instructions. For detection of G-Actin, fixed and permeabilised cells (prepared as above) were stained with G-actin binding deoxyribonuclease 1-Alexa-Fluor-594 (DNAse1) conjugate for 30 minutes. Following staining, cells were washed in PBS and mounted onto glass microscope slides in Slow-Fade Gold Antifade mounting medium (Life Technologies). Images were acquired using an Olympus fluorescence microscope using an oil immersion x50 objective. For quantification of G-actin, TIFF images were converted to inverted grayscale images using Photoshop (Adobe). Total DNAse1 staining pixel intensity per field of view was quantified by densitometry using Quantity One software (Bio-Rad) and divided by the cell number to obtain a value representing DNAse1 staining intensity per cell.

Table 1:

PRIMER SEQEUENCE (5'-3')	PRIMER DESCRIPTION
CAGCTCACTGAAGAGGCTTCCTGG	Rat CCN1 forward
TAGACCGGTGCAGACACACAATGG	Rat CCN1 reverse
ACGAGACACTCCAAACGAAGCCAT	rat CTGF forward
AGTGGCCTTCCTCAACCCTCTGTA	rat CTGF reverse
ATCCAAGCCATCCCACGAAACTCC	rat ACTA2 forward
AGCTGGTGGCCTTTCACAAGAGAG	rat ACTA2 reverse
ACAGCAGAATGCACCCAGAACAGT	rat TPM1 forward
GCTTAGCCCTAAAGTCTGCAGCGA	rat TPM1 reverse
GCCTTTGAGGCGGCTTTTAACCAG	rat 36B4 forward
AGTGACTGACCAGGGAACGAGACT	rat 36B4 reverse
TAAGATGCTTGTGGTTTGGCCCCT	human CCN1 forward
ATGTGCCTTTCTGCCAAGCTACCA	human CCN1 reverse
GACATTCCAAGAGAGGCTCTGGCT	human CTGF forward
GCGACTCTACAAGAGGAGTGGCTT	human CTGF reverse
CCTCAGAAGAGCTCCTGGTGCTTG	human ACTA2 forward
GCCTTCACACCACTGACAGGAACA	human ACTA2 reverse
GGTGATGCACACAGCTAAGGCTCT	human TPM1 forward
ACTTTCCGAGGCAGAAGAGGCAAG	human TPM1 reverse
GTATATGCTGTCAGCCTTGCCCCC	human 36B4 forward
TGAAAACACAGTCCTTGGTTACAGGG	human 36B4 reverse

Table 1: Sequences of qPCR primers used

SUPPLEMENTARY DATA

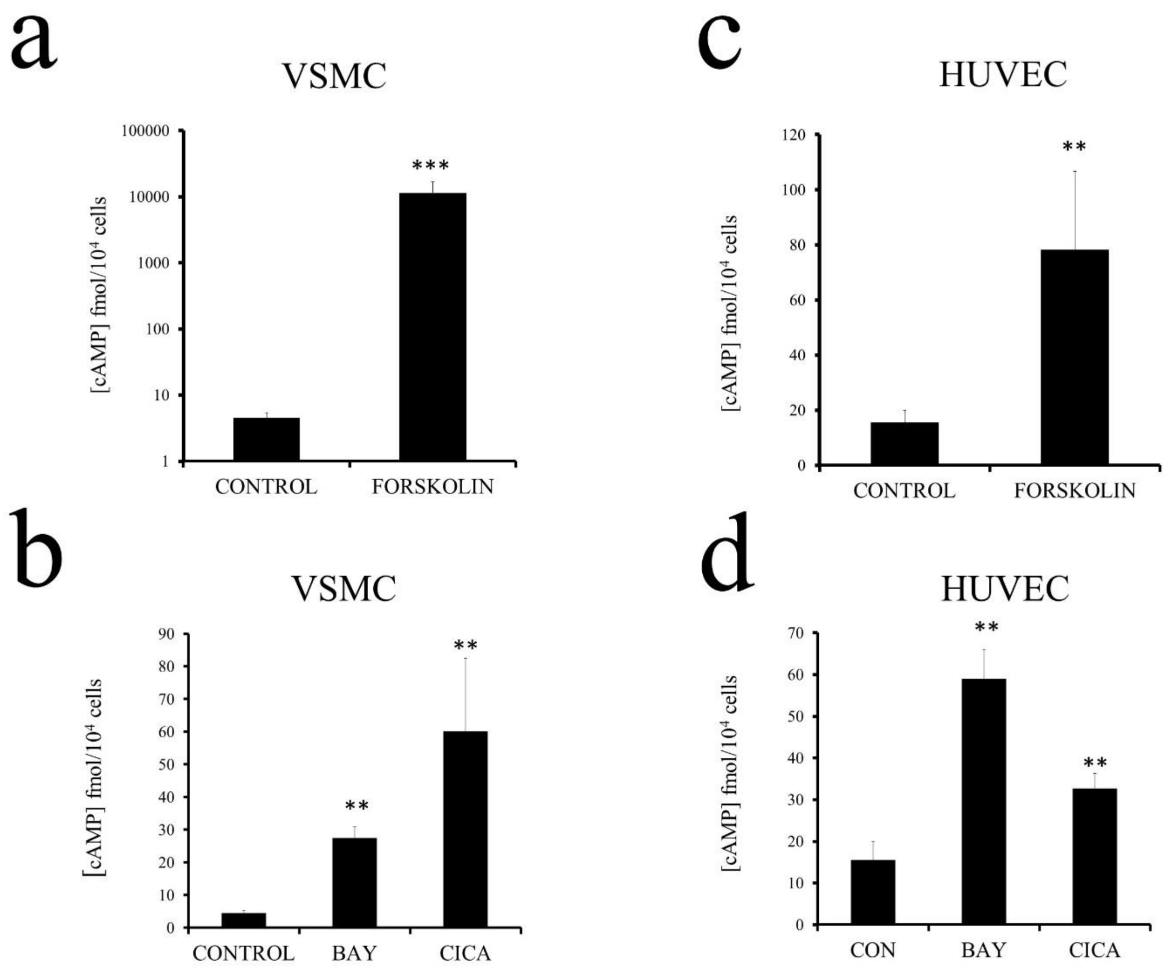

Supplement Figure 1: Quantification of cAMP levels after forskolin, BAY60-6583 or Cicaprost stimulation of RaVSMC and HUVEC.

RaVSMCs (n=3), and HUVECs (n=3) were stimulated for 30 minutes with either 25 µM forskolin, 1 µg/ml BAY60-6583 or 1 µM Cicaprost. Cells were lysed in 0.1 M HCl and cAMP levels quantified by direct cAMP ELISA. ** indicates $p<0.01$; *** indicates $p<0.001$. One-way ANOVA with replication and Student Newman Keuls post-test

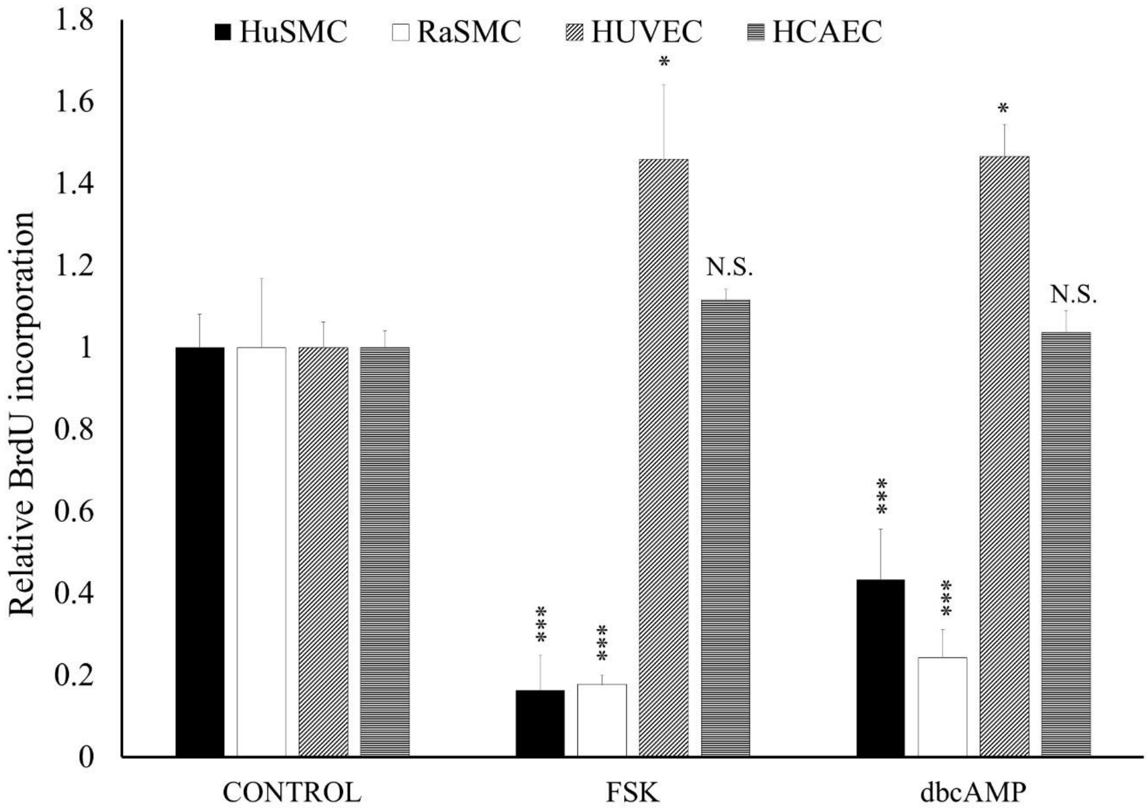

Supplement Figure 2: Elevated cAMP inhibits VSMC but not EC proliferation

RaVSMCs (n=3), HuVSMCs (n=3), HUVECs (n=3) and HCAECs (n=3) were stimulated for 18 hours with either 25 µM forskolin (FSK) or 500 µM db-cAMP, in 10% FCS, as indicated. Cells were labelled with 10 µM BrdU for further 6 hours and proliferation quantified by immuno-histochemical staining of incorporated BrdU. * indicates $p<0.05$, *** indicates $p<0.001$. One-way ANOVA with replication and Student Newman Keuls post-test.

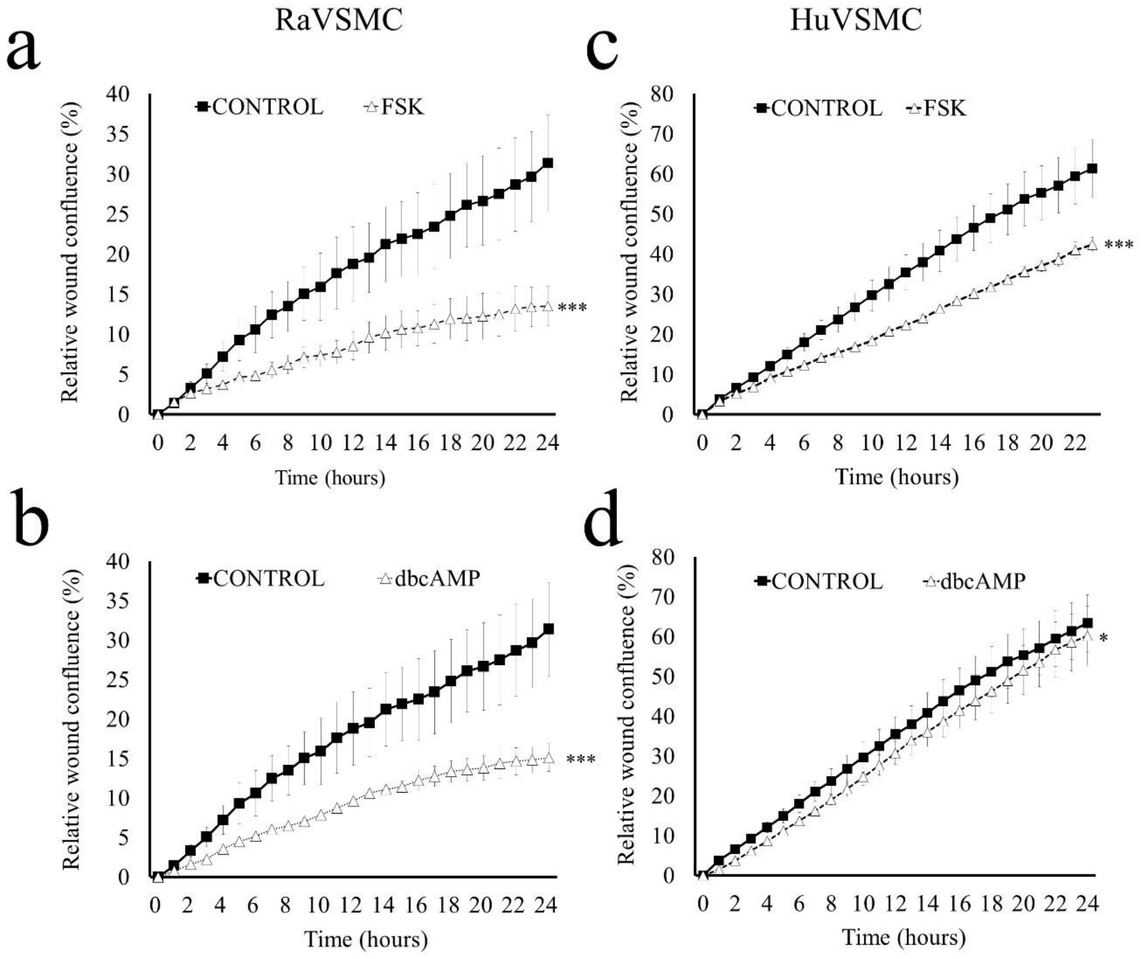

Supplement Figure 3: Elevated cAMP inhibits VSMC migration

Real-time scratch-wound migration analysis of RaVSMCs (A and B; n=3) and HuVSMCs (C and D; n=3) stimulated with 25 µM forskolin (FSK) (A and C) or 500 µM db-cAMP (B and D) was performed using an IncuCyte ZOOM live-cell analysis system. * indicates $p<0.05$; *** indicates $p<0.001$. Two-way ANOVA with replication.

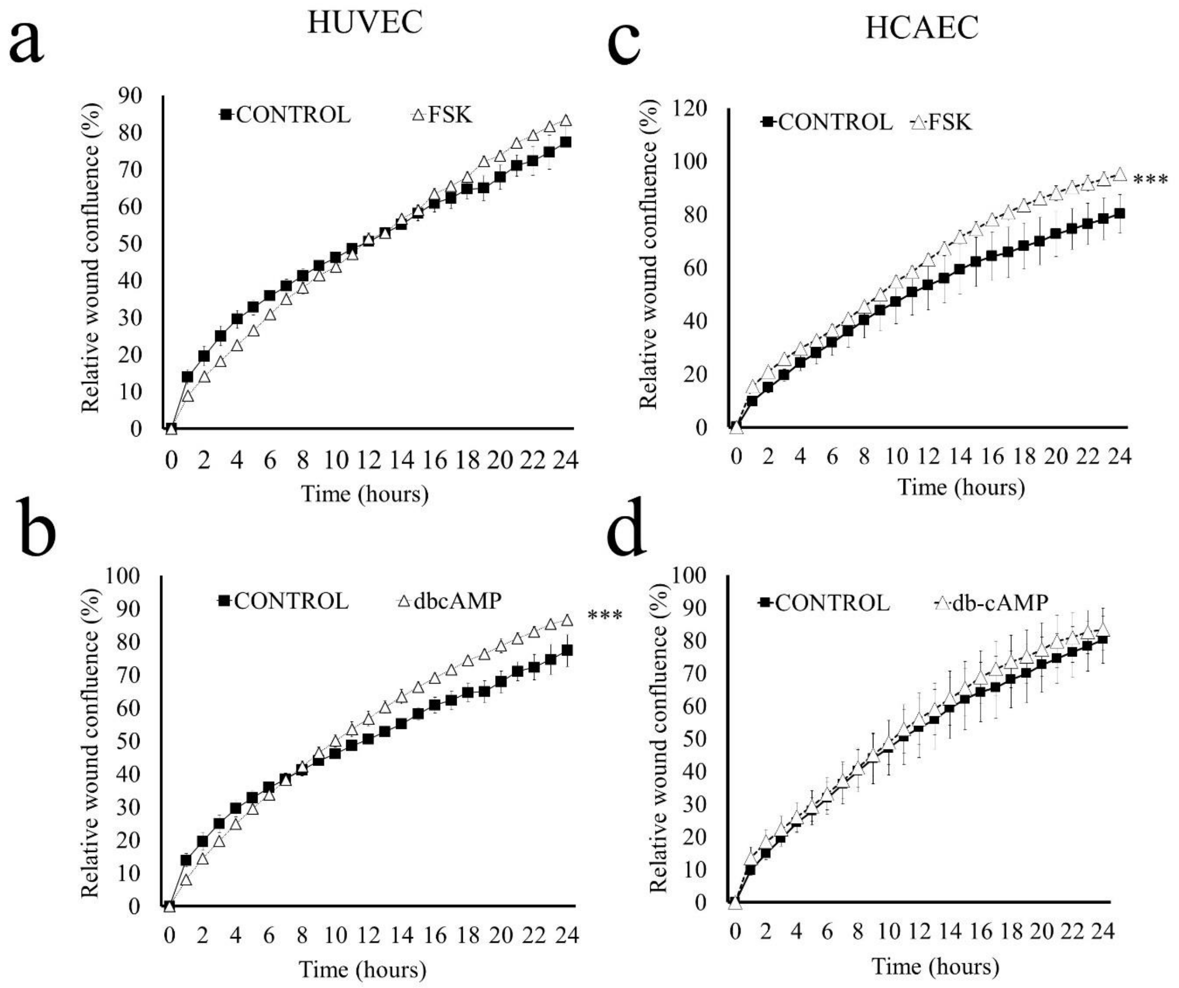

Supplement Figure 4: Elevated cAMP does not inhibit EC migration

Real-time scratch-wound migration analysis of HUVECs (A and B; n=3) and HCAECs (C and D; n=3) stimulated with 25 µM forskolin (FSK) (A and C) or 500 µM db-cAMP (B and D) was performed using an IncuCyte ZOOM live-cell analysis system. * indicates $p<0.05$; *** indicates $p<0.001$. Two-way ANOVA with replication.

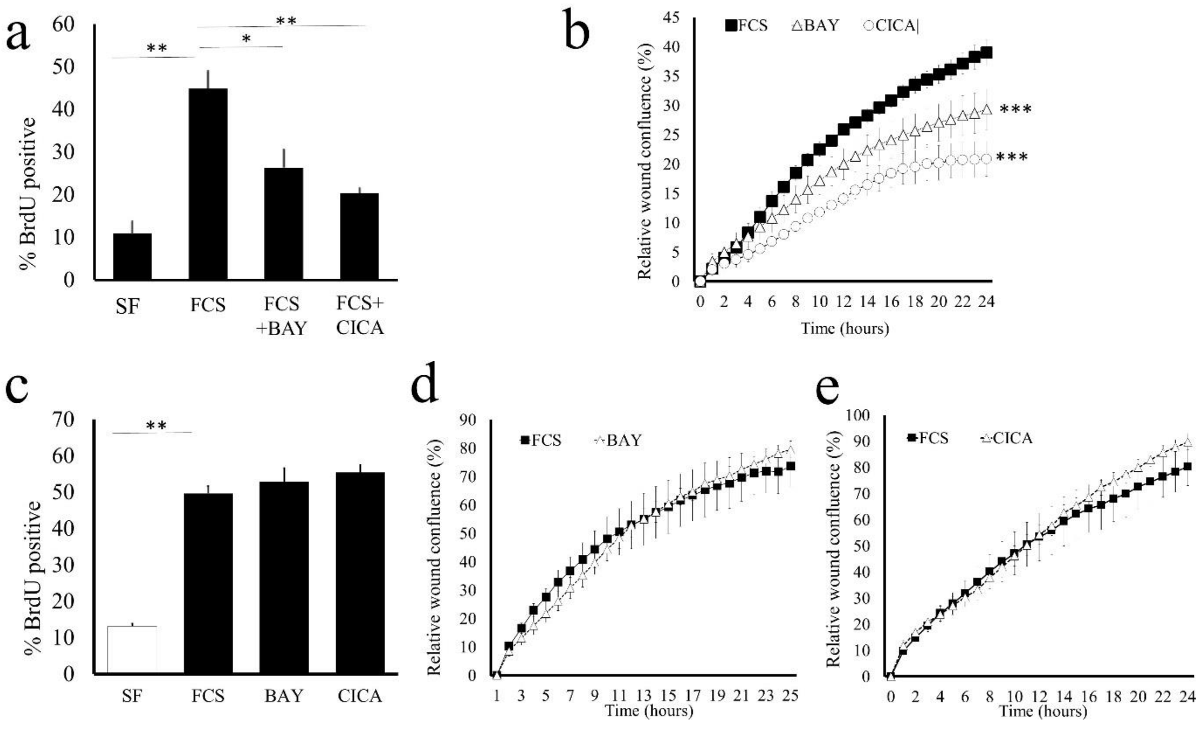

Supplement Figure 5: **BAY60-6583 and Cicaprost inhibit VSMC but not EC proliferation and migration**

RaVSMCs and HCAEC were treated with 1µg/mL BAY 60-6583 (BAY) or 1µM Cicaprost (CICA) for 18 hours and labelled with 10 µM BrdU for further 6 hours. Proliferation was quantified by immuno-histochemical staining of incorporated BrDU (A and C; n=3). Real-time scratch-wound migration analysis of RaVSMCs and HCAEC stimulated with 1µg/mL BAY 60-6583 or 1µM Cicaprost was performed using an IncuCyte ZOOM live-cell analysis system (B, D and E; n=3). * indicates p<0.05, ** indicates p<0.01, *** indicates p<0.001. One-way ANOVA with replication and Student Newman Keuls post-test (A and C). Two-way ANOVA (B, D and E).

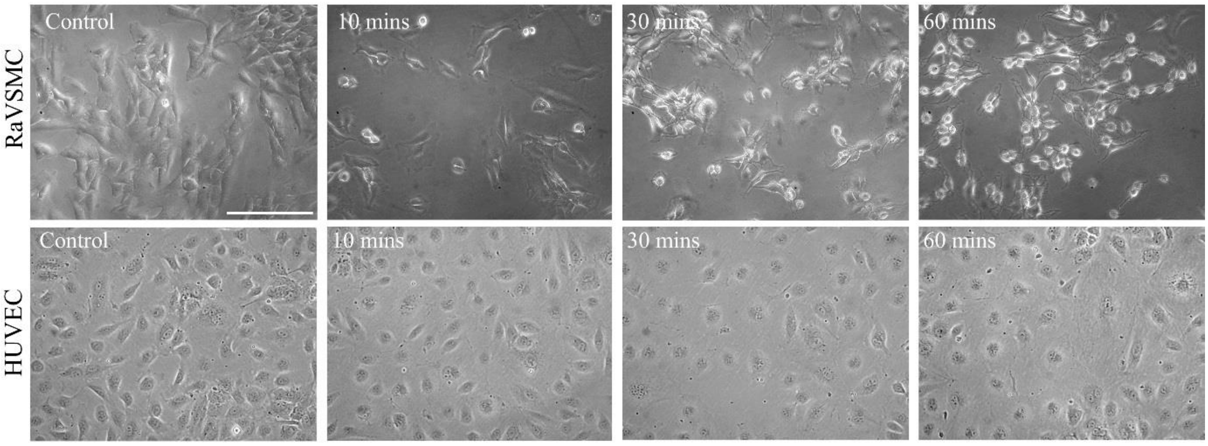

Supplement Figure 6: Forskolin induces morphological changes in VSMCs but not ECs

RaVSMCs and HUVECs were stimulated in serum free conditions with 25 µM forskolin and phase contrast microscopy images taken at the indicated times. Bar indicates 250 µm.

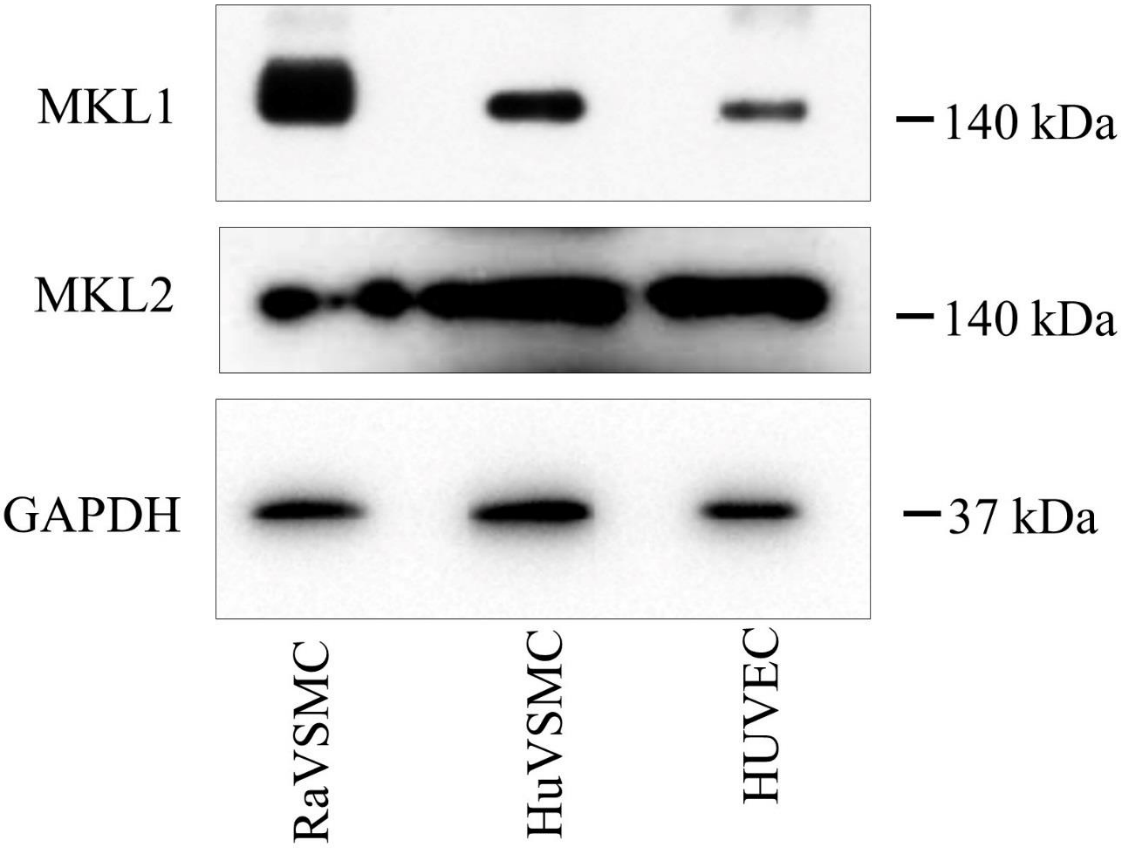

Supplement Figure 7: Endogenous expression of MKL1 and MKL2 in rat VSMC, human VSMC and HUVEC

Total cell lysates were prepared from RaVSMCs, HuVSMCs or HUVECs and analysed for MKL1, MKL2 and GAPDH expression by western blotting. Cropped blots are shown.

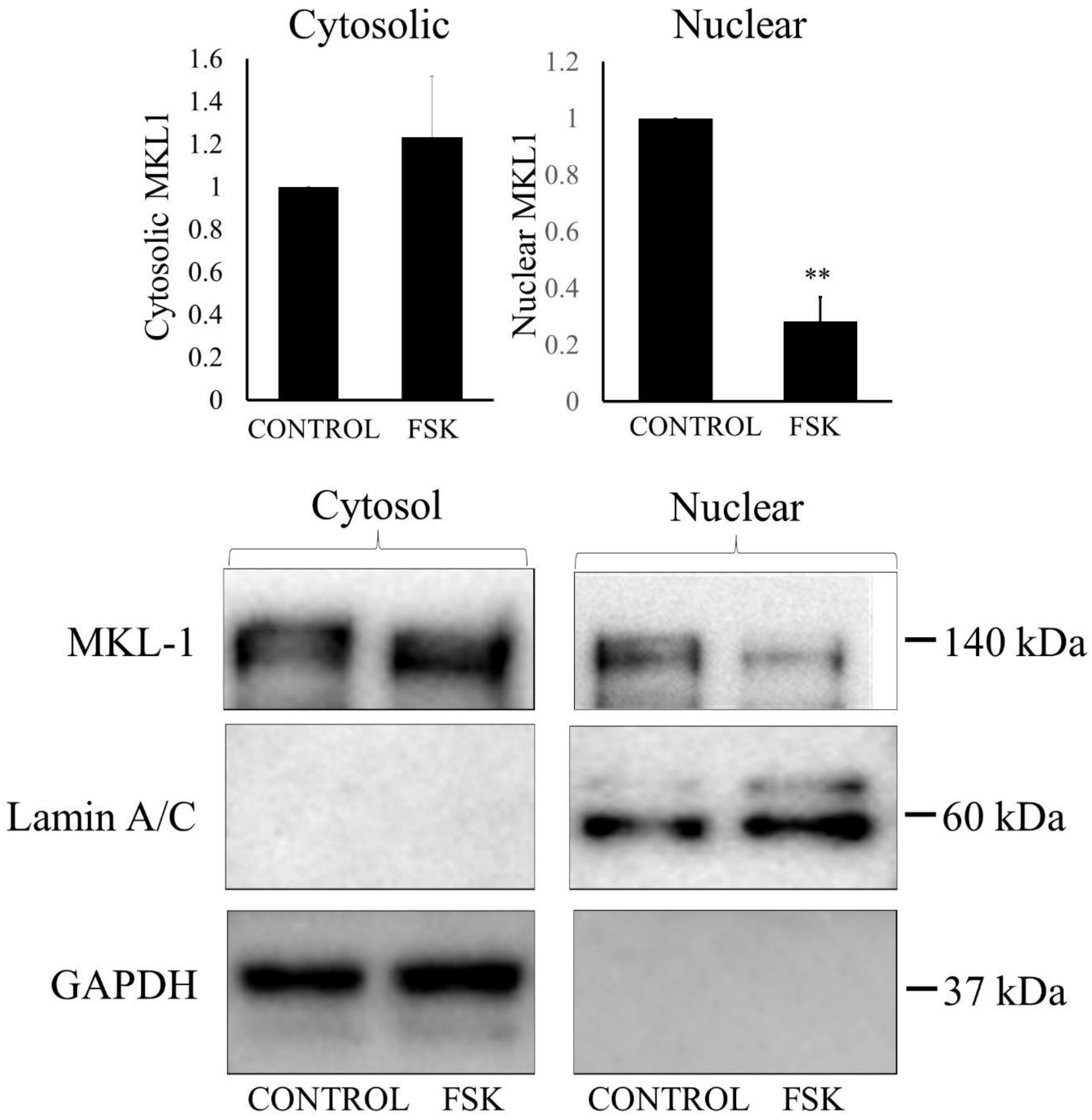

Supplement Figure 8: Forskolin inhibits nuclear localisation of endogenous MKL1 in VSMC

Serum starved RaVSMCs were stimulated with 10% FCS in the presence or absence of 25 µM forskolin (FSK) for 1 hour. Cytoplasmic (n=3) and nuclear (n=4) fractions were prepared and analysed for MKL1. Blots were probed for Lamin A/C (nuclear marker) and GAPDH (cytosolic marker) to confirm efficient fractionation. Cropped blots are shown. ** indicates $p<0.01$. One-way ANOVA with Student Newman Keuls post-test.

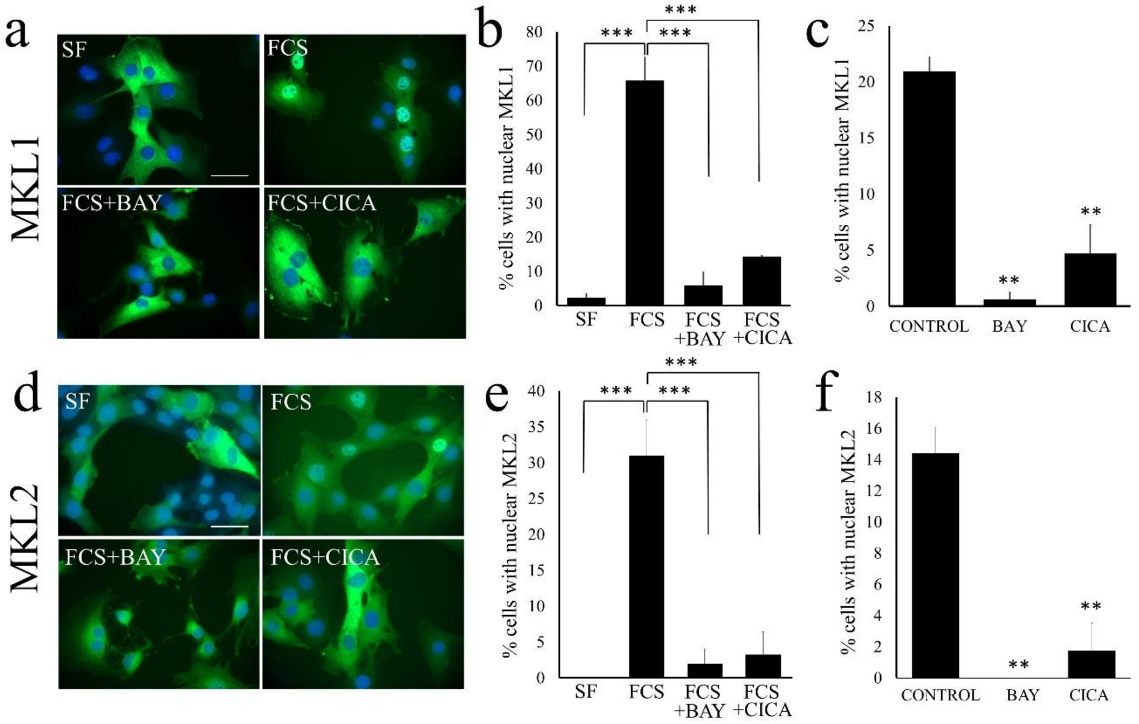

Supplement Figure 9: BAY60-6583 and Cicaprost inhibit nuclear localisation of MKL1 and MKL2 in RaVSMCs

RaVSMCs were infected with adenoviral vectors expressing GFP-MKL-1 (A, B, C; n=3) or GFP-MKL2 (D, E, F; n=3). Asynchronously proliferating VSMC (C, F) or VSMC serum starved for 4 hours (A, B, D, E) were stimulated for 1 hour with 1µg/mL BAY 60-6583 (BAY) or 1µM Cicaprost (CICA), as indicated. Cells were analysed for cellular localisation of MKL1 (A-C) and MKL2 (D-F). ** indicates p<0.01, *** indicates p<0.0001 with respect to nuclear localisation; one-way ANOVA with Student Newman Keuls post-test. Bar indicates 50 µm.

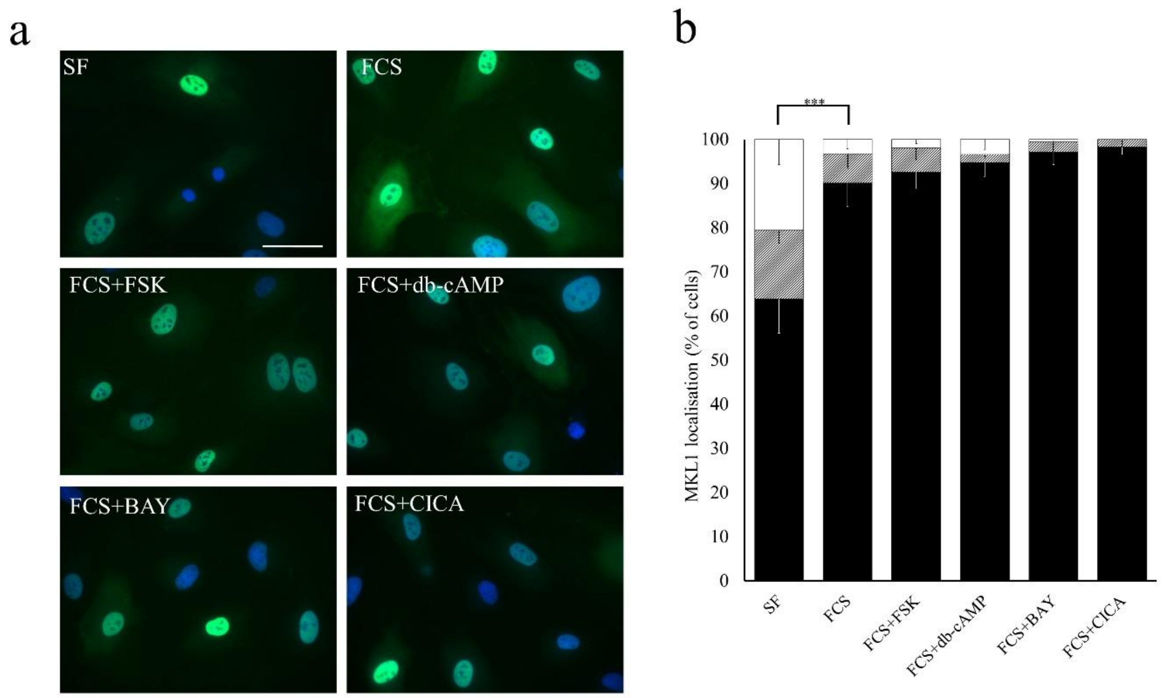

Supplement Figure 10: Elevated cAMP, BAY60-6583 and Cicaprost do not inhibit nuclear localisation of MKL1 in HCAECs

HCAECs were infected with adenoviral vectors expressing GFP-MKL-1. Cells were serum starved for 4 hours before being stimulated for 1 hour with 10% FCS in the presence of either 25 µM forskolin (FSK), 500 µM db-cAMP, 1µg/mL BAY 60-6583 (BAY) or 1µM Cicaprost (CICA), as indicated. Cells were analysed for cellular localisation of MKL1 by fluorescence microscopy (A). Cellular localisation of MKL1 (classified as either nuclear, cytoplasmic or equally distributed between the cytoplasm and nucleus) was quantified by image analysis (B). Images are representative of at least three separate experiments. *** indicates $p<0.0001$ with respect to nuclear localisation; One-way ANOVA with Student Newman Keuls post-test; n=3. Bar indicates 50 µm.

MKL1

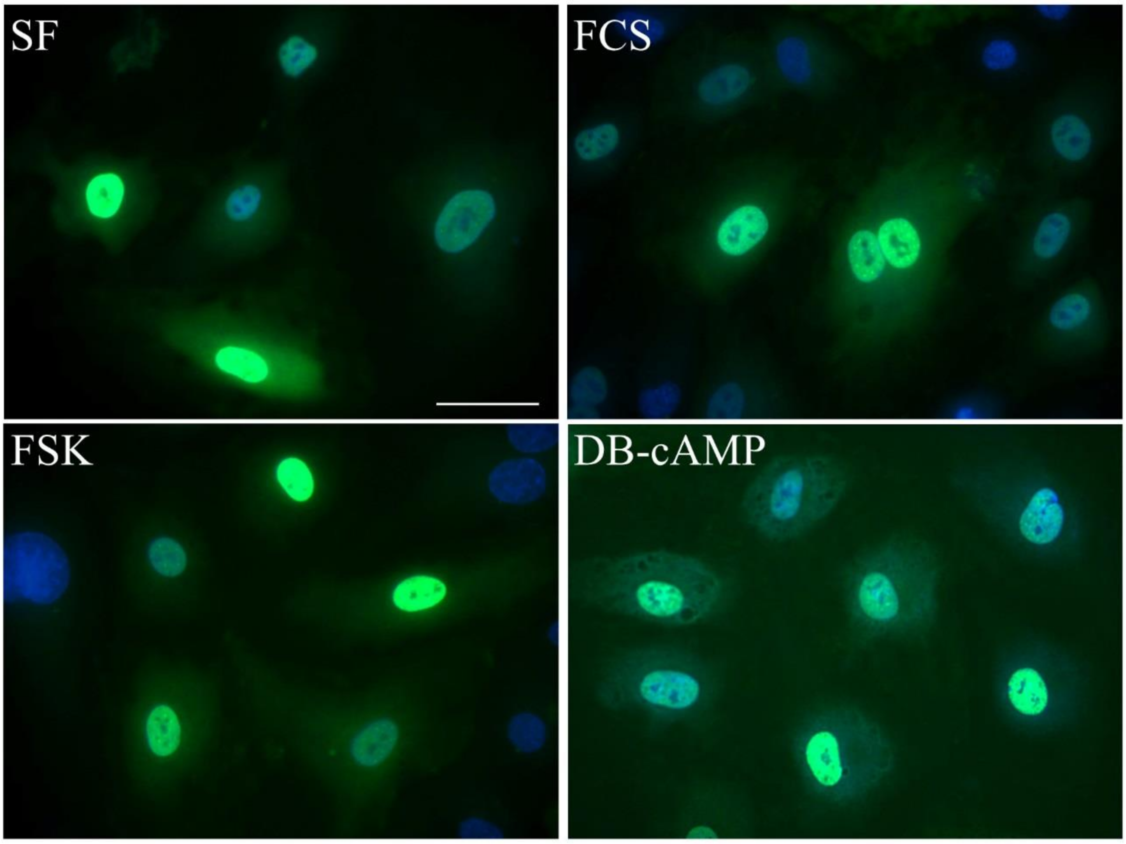

Supplement Figure 11: Prolonged serum starvation and cAMP elevation does not inhibit nuclear localisation of MKL1 in HUVECs

HUVECs were infected with adenoviral vectors expressing GFP-MKL-1. Cells were serum starved for 18 hours before being stimulated for 18 hours with 10% FCS in the presence of either 25 µM forskolin (FSK) or 500 µM db-cAMP, as indicated. Cells were analysed for cellular localisation of MKL1 by fluorescence microscopy. Bar indicates 50 µm.

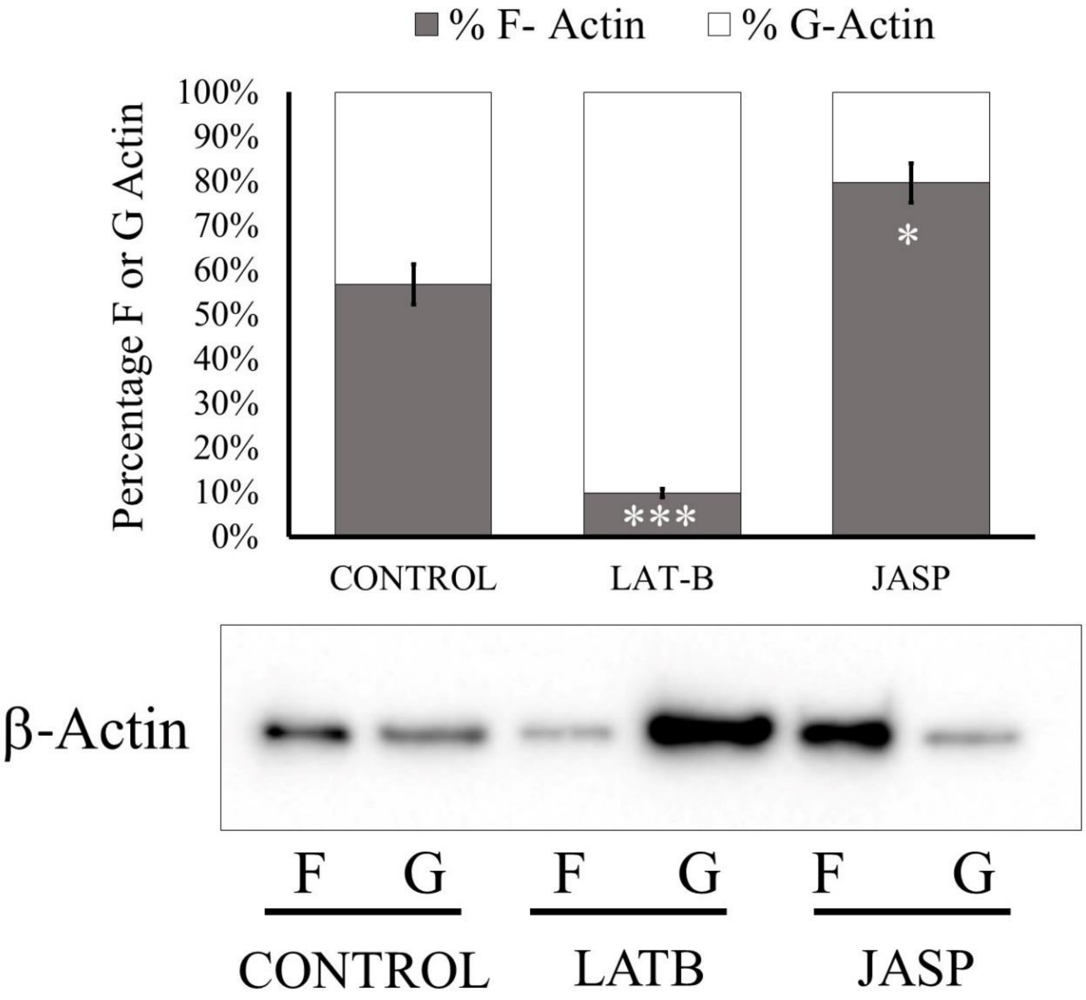

Supplement Figure 12: Quantification of F- and G- actin levels in cells treated with latrunculin-B or Jasplakinolide.

RaVSMC were stimulated with 5 µg/ml latrunculin B (LAT-B) or 1 µM Jasplakinolide (JASP) for 30 minutes. G-actin was solubilised in 0.1 Triton-X-100 and insoluble F-actin extracted in SDS lysis buffer. F- and G- actin levels were quantified by western blotting (n=3). Cropped blots are shown. * indicates $p<0.05$; *** indicates $p<0.001$. One-way ANOVA with Student Newman Keuls post-test.

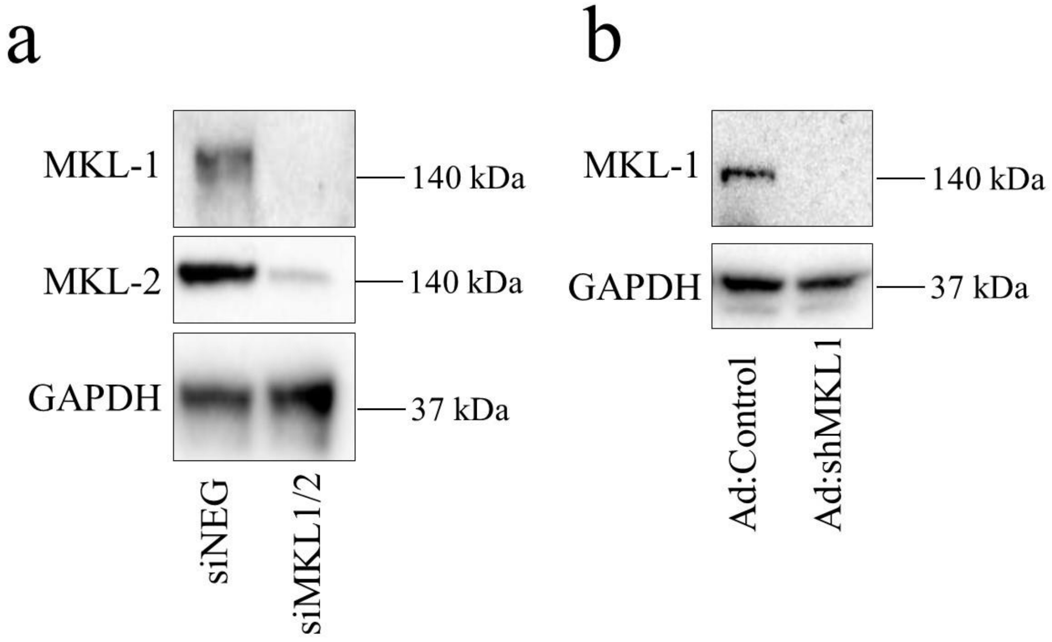

Supplement Figure 13: siRNA and shRNA efficiently silence MKL1 and MKL2 protein levels in VSMCs and HUVECs

VSMCs were transfected with 50 pmoles of siRNA targeting MKL1 and MKL2. Silencer Select (Invitrogen) siRNA negative control was used as a control (A). HUVEC were infected with adenovirus vector expressing shRNA targeting MKL1 (B). Total cell lysates were prepared 48 hours post transfection and analysed by Western blotting for MKL1, MKL2 and GAPDH. Cropped blots are shown.

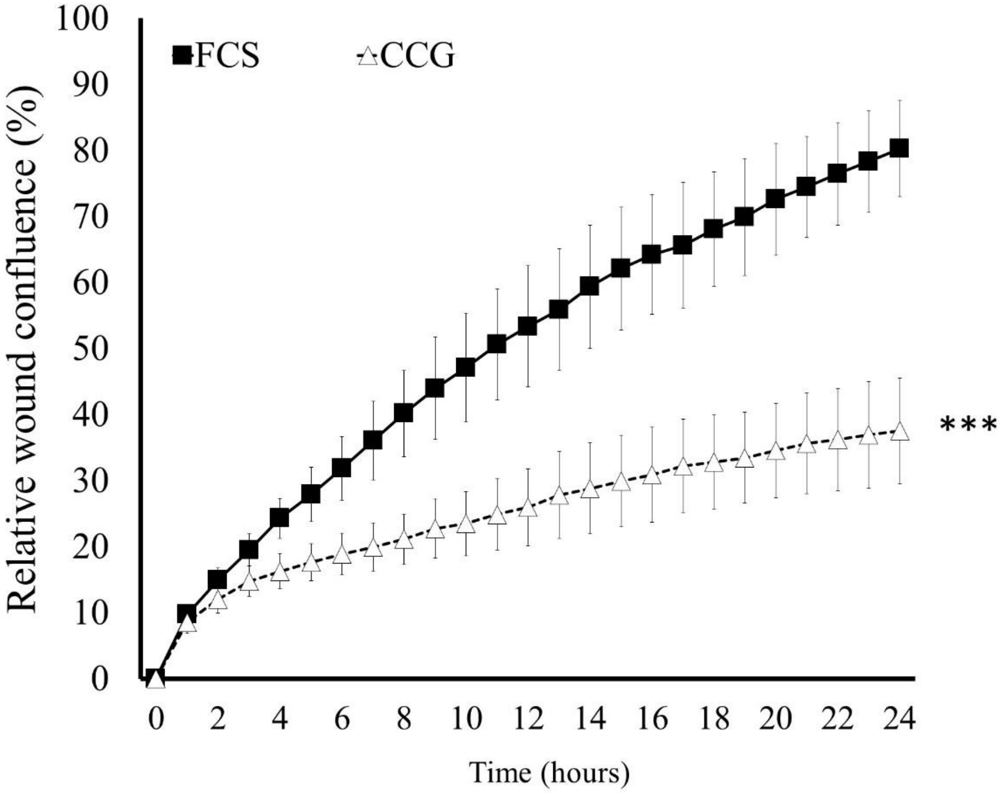

Supplement Figure 14: CCG203971 inhibits HCAEC migration

HCAECs were treated with 20 µM CCG203971 (CCG) in 10% FCS for 24 hours and migration analysed using IncuCyte real-time scratch wound assays (n=3). *** indicates p<0.0001. Two-way ANOVA with replication.

.

FULL LENGTH SCANS OF WESTERN BLOTS

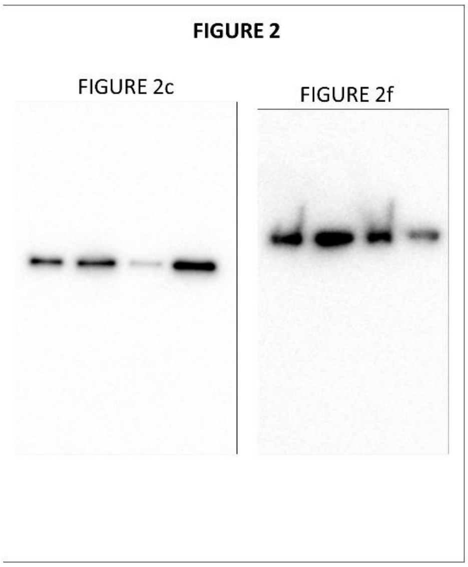

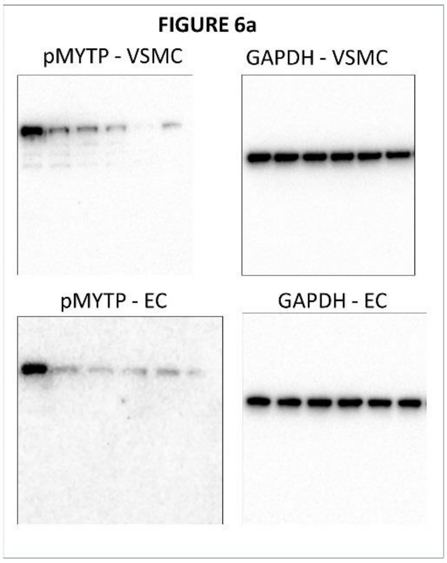

Supplement: Supplementary file 1 — supplementary Info [file 41598_2017_3337_MOESM1_ESM.pdf]
